# A review of the effects of artemether-lumefantrine on gametocyte carriage and disease transmission

**DOI:** 10.1186/1475-2875-13-291

**Published:** 2014-07-28

**Authors:** Michael Makanga

**Affiliations:** 1 European & Developing Countries Clinical Trials Partnership (EDCTP), PO Box 19070, Tygerberg, Cape Town, South Africa

**Keywords:** Artemether-lumefantrine, Gametocytes, Malaria eradication, Systematic review

## Abstract

While significant advances have been made in the prevention and treatment of malaria in recent years, these successes continue to fall short of the World Health Organization (WHO) goals for malaria control and elimination. For elimination strategies to be effective, limited disease transmission, achieved through rapid reduction in the infectious parasite reservoir and decreased gametocyte carriage, will be critical. Artemisinin-based combination therapy (ACT) forms the cornerstone of WHO-recommended treatment for uncomplicated *Plasmodium falciparum* malaria, and in combination with other effective interventions will undoubtedly play a vital role in elimination programmes. The gametocytocidal properties of artemisinins are a bonus attribute; there is epidemiological evidence of reductions in malaria incidence and transmission in African regions since the introduction of these agents. Many studies and analyses have specifically investigated the effects of the ACT, artemether-lumefantrine (AL) on gametocyte carriage. In this systematic review of 62 articles published between 1998 and January 2014, the effects of AL on gametocyte carriage and malaria transmission are compared with other artemisinin-based anti-malarials and non-ACT. The impact of AL treatment of asymptomatic carriers on population gametocyte carriage, and the potential future role of AL in malaria elimination initiatives are also considered. Despite the inherent difficulties in comparing data from a range of different studies that also utilized different diagnostic approaches to assess baseline gametocyte counts, the gametocytocidal effect of AL was proportionately consistent across the studies reviewed, suggesting that AL will continue to play a vital role in the treatment of malaria and contribute to clearing the path towards malaria elimination. However, the specific place of AL is the subject of much ongoing research and will undoubtedly be dependent on different demographic and geographical scenarios. Utilizing ACT, such as AL, within malaria elimination strategies is also associated with a number of other challenges, such as balancing potential increased use of ACT (e g, treatment of asymptomatic carriers and home-based treatment) with rational use and avoidance of drug resistance development.

## Background

### From malaria control to elimination

Between 2000 and 2012 estimated malaria mortality rates across all age groups dropped by 42% and 49% globally and in Africa, respectively, and deaths decreased by 51% in Africa in children < five years of age [1]. Modelling estimates indicate that between 2000 and 2012, 3.3 million malaria deaths were averted, 90% of which were estimated to be in children < five years in sub-Saharan Africa [1]. However, the rate of decline in estimated malaria mortality slowed between 2011 and 2012. This is partially attributed by the World Health Organization (WHO) to insufficient funds to provide insecticide-treated bed nets (ITNs) to countries with ongoing transmission of the disease, and a relatively slow roll-out and interrupted supplies of preventative therapy to children < five years and pregnant women in recent years [1]. This may also have been partly due to ongoing interruptions in the supply chain of artemisinin-based combination therapy (ACT) to populations in need [2,3], as well as the relatively slow global roll-out of intravenous artesunate. Encouragingly, in 2013, 136 million nets were delivered to malaria-endemic countries and this number looks set to increase further throughout 2014 [1]. In addition, increasing access to WHO-recommended ACT has been reported; the number of delivered treatment courses has increased from 76 million in 2006 to 331 million in 2012 [1].

Despite all of the above measures, during 2012 there were an estimated 207 million cases of malaria, resulting in approximately 627,000 malaria deaths. An estimated 3.4 billion individuals remain at risk of malaria, primarily in Africa (where 80% of cases occur) and Southeast Asia [1]. Therefore, while significant advances have been made in managing malaria in recent years, these successes continue to fall short of WHO goals for malaria control and elimination (i e, to reduce global malaria deaths to near zero by end of 2015; to reduce global malaria cases by 75% by end of 2015; and to eliminate malaria by end of 2015 in ten new countries since 2008, including in the WHO European Region) [1].

A WHO global technical strategy for malaria control and elimination is currently planned for the period 2016 to 2025, in addition to a global plan to control and eliminate *Plasmodium vivax* malaria [4]. Moreover, several other public health intervention strategies are also being implemented [5-7]. As countries move towards malaria elimination, limiting transmission of the disease will be critical. In order to achieve this, treatment will need to be able to rapidly reduce the infectious parasite reservoir, decrease gametocyte carriage and thus reduce infectiousness to mosquitoes.

### *Plasmodium* life cycle and the role of gametocytes in transmission

**Figure 1:**
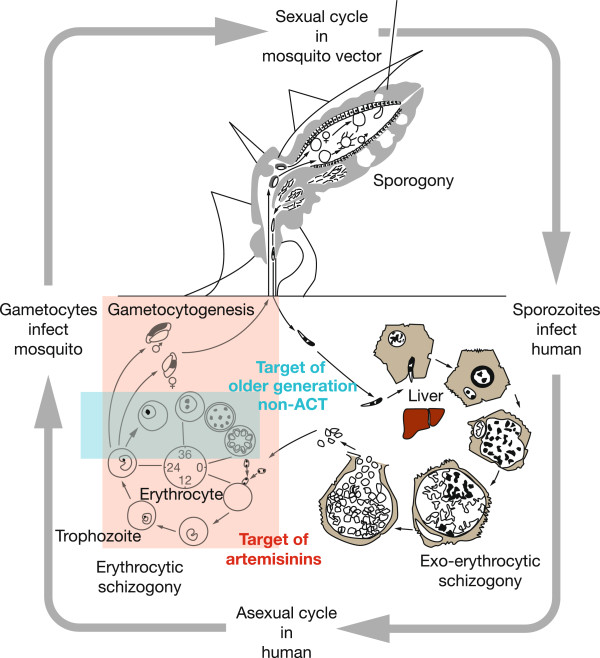
**Life cycle of *Plasmodium falciparum.*** Artemisinin acts at an early stage of the erythrocytic stage of the parasite’s life cycle and is also effective against the gametocyte stage, thereby potentially interrupting transmission of malaria [8]. (Sourced from Hommel M 2008, with permission [8]).

The *Plasmodium* life cycle and targets of ACT and older generation, non-ACT anti-malarials are illustrated in Figure 1[8]. Key to the transmission of malaria is the differentiation within red blood cells of a small number of haploid asexual parasites into male and female gametocytes. Following their ingestion by the mosquito, these male and female gametes fuse to form a zygote and then an oocyst. Multiple rounds of DNA replication result in the production of thousands of sporozoites that are transmitted to humans via the mosquito’s salivary glands, and the cycle begins again. Gametocytes are also integral to the transmission and propagation of disease resistance; compared with drug-sensitive infections, resistant infections are associated with increased rates of recrudescence and slower initial treatment responses, both of which increase gametocyte densities. This suggests that increased gametocyte carriage in infections caused by resistant parasites results in a transmission advantage that ultimately helps to drive the spread of resistance [9,10].

Several factors are likely to influence the appearance of gametocytes at presentation, including age, host immune response (including co-infection with other pathogens), host anaemia, insecticide spraying, and mass drug administration [11]. Risk factors that have been found to be specifically associated with gametocytaemia after treatment in studies include trimethoprim-sulphamethoxazole (TS) prophylaxis [12], increased age (in children) [12], recurrent parasitaemia [12], duration of parasitaemia [13], the presence of any degree of anaemia [13,14], gametocytaemia at enrolment [15], and treatment with artesunate (AS) or artesunate-mefloquine (AS-MQ) compared with other ACT [artemether-lumefantrine (AL), Coartem^®^, or artesunate-amodiaquine (AS-AQ)] [15].

While gametocyte carriage is integral to the transmission of malaria, it is important to note that their presence and detection in peripheral blood does not necessarily equate to infectivity. In this respect, although there is an overall positive correlation between gametocyte density and the mosquito infection rate, this correlation is not particularly strong. One reason for this is likely to be due to the presence of immature gametocytes. Another reason may be due to the poor sensitivity of microscopy for detecting gametocytes. In this regard, gametocytes are invariably detected in fewer than 50% of clinical and asymptomatic *Plasmodium falciparum* infections. In fact, the gametocytes are likely to be present in the majority of infections, just at low densities that evade microscopic detection [11]. This point is further discussed in this review.

### Artemisinin-based combination therapy

ACT forms the cornerstone of WHO-recommended treatment for uncomplicated *P. falciparum* malaria [16], and will undoubtedly play a vital role in malaria elimination strategies [7]. An important feature of artemisinin derivatives, that complements their high efficacy against asexual plasmodium parasites, is their gametocytocidal properties; ACT can shorten the duration of gametocyte carriage by approximately four-fold [17]. There is epidemiological evidence of reductions in malaria transmission and incidence in various African regions since the introduction of ACT [18] when used together with indoor residual spraying and ITNs. In a pooled analysis of six randomized trials, patients treated with ACT had significantly lower gametocytaemia than non-ACT treatment during 28 days of follow-up, and this was associated with reduced human to mosquito transmission [19]. Similarly, in a study in Northwest Thailand, gametocyte reduction following ACT translated into a six-fold decrease in parasite transmission (reviewed in [18]). Field studies have shown that artemisinin treatment significantly reduced gametocyte carriage compared with other agents and this effect was variable depending on geographical region, level of malaria endemicity and access to treatment [8].

AL, which was originally developed and investigated in China [20], was approved by Swissmedic in 1999 and by the U.S. Food and Drug Administration in 2009 for the treatment of uncomplicated *P. falciparum* malaria. There is now extensive clinical experience with AL in patients of all ages and from many regions of the world, and it is one of the most widely used ACT. In addition to the key clinical trials that led to the drug’s approval, numerous independent studies (>60, including >12,000 subjects) have supported the clinical efficacy of this agent, and many studies and analyses have specifically investigated the effects of AL on gametocyte carriage.

This systematic review describes the effects of AL on gametocyte carriage and transmission of malaria, and compares the effects of AL with other artemisinin-based anti-malarials. It also discusses the impact of AL treatment of asymptomatic carriers on population gametocyte carriage, and considers the potential future role of AL in malaria elimination strategies.

## Methods

### Search strategy and selection criteria

References for this review were identified through searches of the following databases that were conducted on 12 September, 2013 for the period 1995 to 2013, and on 31 January, 2014 for the period 12 September to 31 January 2014: Embase (1996 to 10 September, 2013), BIOSIS Previews (1995 to January 2013), EBM Reviews - Cochrane Database of Systematic Reviews (2005 to December 2011), Ovid MEDLINE^®^ without Revisions (1996 to August 2013), Ovid MEDLINE^®^ In-Process & Other Non-Indexed Citations (January 2012), Ovid MEDLINE^®^ Daily Update (January 2012), Ovid, PubMed and ClinicalTrials.gov (September 2013 to January 2014). The search terms Coartem or Riamet or CGP56697 or CGP 56697 or CGP-56697 or co-artemether or exafal, artemether or artemeter, lumefantrine or benflumelol or benflumetol, gametocyte, and combinations of these terms were used to identify articles relating to AL and/or ACT and gametocyte clearance/carriage. The search was limited to humans and articles published in English. All resulting records were screened and full-text articles read to determine whether they included relevant gametocyte data.

Information extracted from each article included study type and year, geographic location, study population (number of children and/or adults), intervention evaluated, gametocyte diagnostic method, sampling schedule for gametocyte data, key data regarding gametocyte carriage and malaria transmission, and key conclusions regarding effects of AL (and other interventions as appropriate).

The limitations of analysing data from clinical trials that involved population groups of different ages, immune status, number of doses of AL treatment regimens, formulations, malaria transmission patterns, endemicity, study designs as well as diagnostic approaches, are recognized and taken into account in the categorization of related datasets and interpretation of the inferences from the review.

## Results

**Figure 2:**
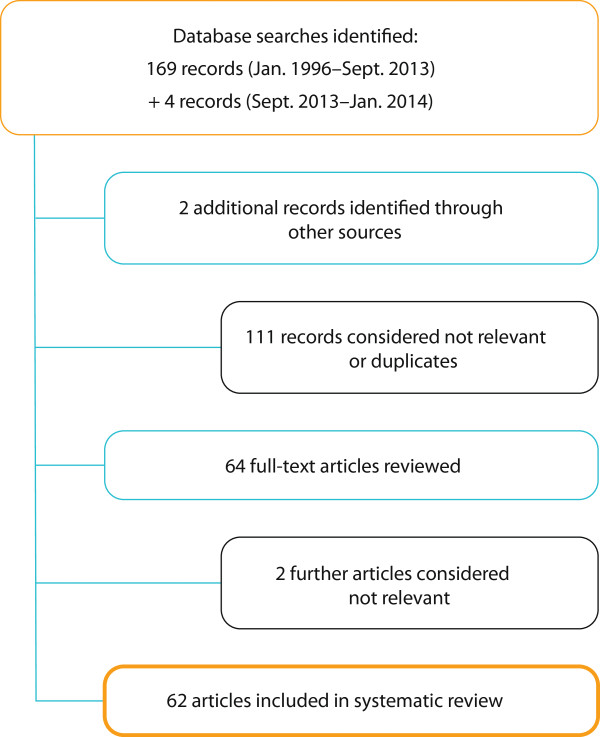
Overview of number of records identified, discarded and included within the systematic review.

From a total of 175 records initially identified, more than half were considered not relevant (i e, did not include data on gametocyte clearance/carriage) or were duplicates, leaving 62 relevant articles for review (Figure 2). These were published between 1998 and January 2014 and included randomized, double-blind and randomized open-label trials (phases II–IV), non-interventional observational studies, single-/multi-centre reports, retrospective analyses of previously conducted efficacy studies, pooled analyses, previously published systematic reviews, reviews, and mini-reviews.

Of the 62 records identified, most (38) reported data for children (aged four months to 15 years), 17 included data for both children and adults, and four for adults only (the three additional reports were general reviews). The majority (45) of studies reported data for African countries. Other study locations included Asia, Thailand, India, Cambodia, Colombia, and Europe. Most reports (42) noted the use of microscopy alone to detect gametocytes. An additional 12 reports utilized both microscopy and polymerase chain reaction (PCR) techniques or compared one approach with the other, and three used PCR alone (in the remaining five reports, details of the gametocyte diagnostic technique were not stated or not applicable). Five of the reports (encompassing three studies in two settings) also included the use of mosquito feeding assays to detect gametocyte infectiousness to mosquitoes.

Key details relating to all studies are listed in Additional file 1[8,9,11-15,17-19,21-72].

### Effects of artemether-lumefantrine on gametocyte clearance and malaria transmission

Studies that report the effects of AL (in the absence of a comparator treatment) on gametocyte carriage/clearance are reviewed below and summarized in Table 1[28,30,37,47,53,54,62,64,68,70] (and Additional file 2[28,30,37,47,53,54,62,64,68,70]). Unless otherwise stated, all data discussed relate to patients with uncomplicated *P. falciparum* malaria.

**Table 1:** Effects of artemether-lumefantrine on gametocyte carriage/clearance - summary of key conclusions*

**Reference**	**Study description**	**Study population**	**Key conclusions**
Makanga *et al.*[30]	Pooled analysis of 7 studies conducted between 1996-2007	Pooled population: 647 adults and 1,332 children	AL showed high cure rates and rapid resolution of parasitaemia, fever, and gametocytaemia in adults and children
Gbotosho *et al.*[28]	Anti-malarial efficacy studies in Ibadan, southwestern Nigeria	2,585 children aged 0.5-15 years	AL reduced the rate of gametocyte carriage in children with acute falciparum infections at presentation and shortened the duration of male gametocyte carriage after treatment
Assefa *et al.*[37]	28-day therapeutic efficacy study in Kersa District, Addis Ababa	90 adults and children	The study showed a rapid decline in gametocytes with treatment
			The clearance rate was more rapid than that found in other studies, which reported the presence of gametocytes up to day 14 and beyond
John *et al.*[47]	Kipsamoite (7 villages) and Kapsisiywa (9 villages) in the Nandi Hills district of Kenya	8,094 adults and children	Treatment with AL (combined with IRS) reduced gametocyte carriage and density in children compared with the period prior to its implementation
Hatz *et al.*[54]	Open-label, non-comparative study in Europe and non-endemic regions of Colombia	165 non-immune adult travellers	Treatment with AL was effective in clearing gametocytes by end of study in non-immune adults
Juma *et al.*[53]	Randomized, controlled, open-label study comparing AL tablets with AL paediatric suspension in Western Kenya	245 children	AL tablets and the 3-dose suspension effectively cleared gametocytes in these children
Makanga *et al*. [64]	Pooled analysis of 8 studies to compare 6-dose with 4-dose AL regimen	544 children	The 6-dose regimen is associated with a more rapid clearance of parasites and a faster and more sustained reduction in gametocyte carriage than the 4-dose regimen
	4 studies in Africa		
	4 studies in Thailand		
Chanda *et al.*[62]	Open label, one-arm prospective evaluation of paediatric suspension of AL in Zambia	91 children (<10 kg)	AL paediatric suspension was associated with a significant and rapid reduction in gametocytes
Barnes *et al.*[68]	Open-label *in vivo* study in KwaZulu-Natal province, South Africa, to determine therapeutic efficacy of a 6-dose regimen of AL	100 adults	AL contributed to a marked and sustained decrease in malaria cases, admissions, and deaths, by greatly improving clinical and parasitological cure rates and reducing gametocyte carriage
Lefèvre *et al.*[70]	Randomized, open-label, parallel group 4-week trial in Thailand	219 adults and children with multidrug-resistant *P. falciparum* malaria	AL rapidly cleared gametocytes in multidrug-resistant *P. falciparum* malaria

Further data on these studies can be found in Additional file 2.

AL: artemether-lumefantrine; IRS: indoor residual spraying.

*References for this review were identified through searches as documented in the Methods section of this publication. Information extracted from each article included study type and year, geographic location, study population (number of children and/or adults), intervention evaluated, gametocyte diagnostic method, sampling schedule for gametocyte data, key data regarding gametocyte carriage and malaria transmission, and key conclusions regarding effects of AL.

**Figure 3:**
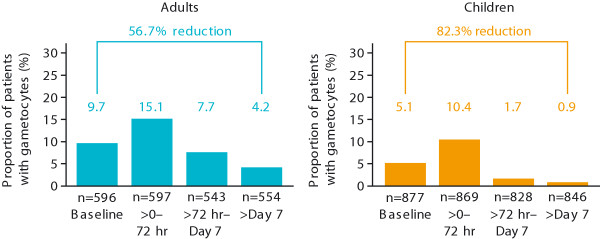
**Reduction in gametocyte carriage with artemether-lumefantrine in adults and children with uncomplicated *Plasmodium falciparum *malaria [**[30]**].** Giemsa-stained thick blood smears were examined for gametocytes. AL: artemether-lumefantrine; mITT: modified intention-to-treat.

Rapid clearance of gametocytes has been demonstrated following treatment with AL in several geographic regions in adults and children. In one of the earliest studies to report the gametocytocidal properties of AL [70], the median time to gametocyte clearance following treatment with AL was 72 hours (three days). The largest dataset evaluating AL therapy to date is provided by a pooled analysis of seven studies conducted over the period 1996 to 2007 involving 1,332 children and 647 adults [30]. Six of the studies were conducted in malaria-endemic areas (four in Thailand and two in Africa) [70,73-77], and the other in non-immune adult travellers in Europe and non-endemic regions of Colombia [54]. Treatment with AL was associated with a rapid reduction in (microscopically determined) gametocyte carriage (from 5.1% of children with gametocytes at baseline to 0.9% after day 7, and from 9.7% of adults at baseline to 4.2% after day 7) (Figure 3) [30].

In the study of 165 non-immune travellers in Europe and non-endemic regions of Colombia [54], the proportion of adults with gametocytes was reduced following treatment with AL so that no patient had gametocytes after day 7. An earlier pooled analysis of eight studies [64] suggested a more rapid clearance of gametocytes following treatment with a six-dose AL regimen than with a four-dose regimen in 544 children ≤12 years; a lower proportion of patients with gametocytes was observed up to day 7 following the six-dose regimen and this effect seemed to be maintained at day 28 (0.3% of patients receiving the six-dose regimen *vs* 3% on the four-dose regimen had circulating gametocytes at days 15 to 28; p < 0.05).

The above data are supported by other observations in children and adults, as detailed in Table 1[28,30,37,47,53,54,62,64,68,70].

### Effects of artemether-lumefantrine compared with other anti-malarials on gametocyte carriage and malaria transmission

#### ***Artemether-lumefantrine versus dihydroartemisinin-piperaquine***

There have been mixed reports regarding the comparative effect of AL and dihydroartemisinin-piperaquine (DP) on post-treatment gametocyte carriage. Key findings are summarized in Table 2 [12,26,32,44,50,52,57] (and Additional file 3[12,26,32,44,50,52,57]).

**Table 2:** **Effects of artemether-lumefantrine *versus *** **dihydroartemisinin-piperaquine on gametocyte carriage/clearance**

**Reference**	**Study description (location)**	**Study population**	**Gametocyte diagnostic method**	**Key conclusions**
Kakuru *et al.*[12]	Open-label randomized controlled trial (AL *vs* DP + TS prophylaxis) in Tororo district, Uganda	351 children (aged ≥4 months)	Microscopy	Rate of gametocyte clearance was more than 2-fold greater with AL than DP (HR 2.20; p < 0.001)
		100 HIV-unexposed		
		203 HIV-exposed		
		48 HIV-infected		
Sawa *et al*. [26]	Randomized, open label trial (AL *vs* DP) in Mbita, Western Kenya Use of QT-NASBA and mosquito feeding assays	298 children (aged 6 months to 10 years)	QT-NASBA, feeding assays	AL was associated with a significantly shorter duration of gametocyte carriage, and a significantly shorter time to gametocyte clearance than DP
				Malaria transmission to mosquitoes was significantly lower after AL treatment than after DP
Smithuis *et al.*[32]	Open-label randomized trial (comparison of ACT, including DP *vs* AL) in Myanmar	>800 adults and children	Microscopy	Gametocyte carriage was variable following treatment with different ACT, although all rates were higher with DP than other ACT regimens, including AL
Zwang *et al.*[44]	Analysis of 7 open-label randomized comparative studies (DP *vs* AL and *vs* other ACT) in Northwest Thailand, Rakhine state, Myanmar, Southern Laos, and Western Cambodia	3,547 adults and children	Microscopy	Clearance of gametocytaemia was slower in DP groups than in the comparators, overall and in individual sites
Yeka *et al.*[50]	Randomized study (AL *vs* DP) in Western Uganda (area of moderate transmission)	408 children (aged 6 months to 10 years)	Microscopy	Patients treated with DP had a lower risk of developing gametocytaemia than those treated with AL after therapy
Mens *et al.*[52]	Randomized study (AL *vs* DP) in Mbita, Western Kenya, use of QT-NASBA	146 children	Microscopy	A more rapid reduction in gametocytes was observed with AL than with DP
			QT-NASBA	
				QT-NASBA provides a far more sensitive method than microscopy in gametocyte detection
Kamya *et al.*[57]	Randomized single-blinded study (AL *vs* DP) in Apac, District, Uganda (area of high transmission)	417 children (aged 6 months to 10 years)	Microscopy	Patients treated with DP had a lower risk of recurrent parasitaemia due to non-falciparum species, and development of gametocytaemia compared with patients treated with AL

Further data on these studies can be found in Additional file 3.

ACT: artemisinin-based combination therapy; AL: artemether-lumefantrine; DP: dihydroartemisinin-piperaquine; HR: hazard ratio; QT-NASBA: quantitative real-time nucleic acid sequence-based amplification; TS: trimethoprim-sulphamethoxazole.

DP was reported to be superior to AL in reducing gametocytaemia, with a longer duration of microscopically detected gametocyte carriage reported after AL than DP in two randomized studies (from the same group) of 408 and 417 Ugandan children in areas of moderate [50] and high transmission intensity [57], respectively. However, data from other studies suggest a similar or shorter duration of gametocyte carriage after treatment with AL than with DP. In an analysis of seven randomized trials, treatment with DP was compared with AL and other ACT [44]. Clearance of gametocytaemia (microscopically determined) was slower with DP than the other agents, both overall and within individual sites. At day 3 following treatment with DP (n = 211), 7.4% of patients still had gametocytaemia compared with 1.8% of patients treated with AL (n = 210). A large longitudinal clinical trial of 351 Ugandan children (≥ four months; 100 HIV-unexposed, 203 HIV-exposed and 48 HIV-infected) reported an 85% increased risk of microscopically determined gametocyte carriage with DP compared with AL during the 28 days following treatment [12]. The rate of gametocyte clearance was more than two-fold greater with AL than DP (hazard ratio (HR) 2.20; p < 0.001) after adjusting for prophylaxis with TS, age and recurrent parasitaemia.

In a comparison of ACT (AL, AS-MQ, AS-AQ, and DP) in >800 adults and children from Myanmar, gametocyte carriage rates varied widely following treatment with the different regimens, although all were lower than with DP. Notably, further reductions (approximately 12 times lower) in gametocyte carriage rates were observed with all ACT regimens following the addition of primaquine [32].

The variation in post-treatment gametocyte carriage across studies may be due to different types of analyses and also the low sensitivity of microscopy for detection of gametocytes; it is now well acknowledged that only a fraction of gametocytes can be detected by this method [11]. For example, a randomized study of 146 Kenyan children previously utilized quantitative real-time nucleic acid sequence-based amplification (QT-NASBA) to evaluate the efficacy and effectiveness of AL *vs* DP in the treatment and control of malaria transmission. Notably, gametocytes were detected in a significantly higher proportion of patients than were detected by microscopy alone and a significantly greater risk of gametocyte carriage was reported after DP than AL treatment [52].

**Figure 4:**
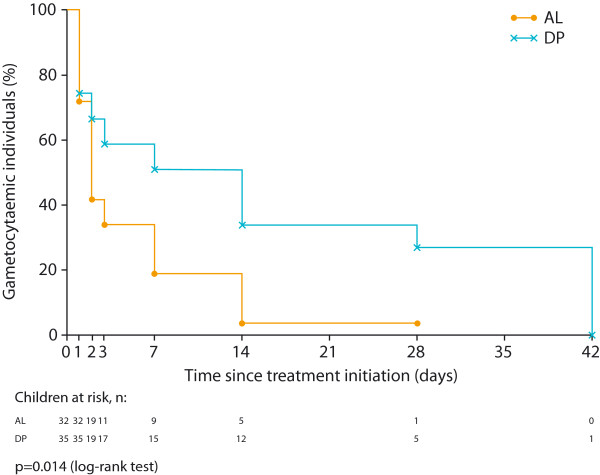
**Time to disappearance of gametocytes in gametocyte-positive individuals (by quantitative real-time nucleic acid sequence-based amplification) at enrolment following treatment [**[26]**].** AL: artemether-lumefantrine (n = 32); DP: dihydroartemisinin-piperaquine (n = 35). (Sourced from Sawa *et al*. 2013, with permission [26]).

Further evidence of the importance of the gametocyte diagnostic method is provided by a randomized trial in 298 African children (aged six months to ten years) who were treated with AL or DP. A highly sensitive molecular assay was used to assess gametocyte carriage, and mosquito-feeding assays were used to determine infectiousness to mosquitoes [26]. The gametocytocidal effect of AL immediately after treatment was larger than that of DP. There was no difference between treatment arms in enrolment gametocyte prevalence (9.7% by microscopy and 71.3% by QT-NASBA). AL was associated with a significantly shorter mean duration of gametocyte carriage than DP (5.5 *vs* 15.3 days, respectively; p = 0.001). For individuals who were gametocyte-positive (by QT-NASBA) prior to treatment, the time to disappearance of gametocytes was significantly shorter for the AL group *vs* the DP group (HR 2.35) (Figure 4) [26]. In terms of gametocyte transmission to mosquitoes that fed on post-treatment blood samples, 1.9% (43/2293) became infected from AL-treated children and 3.5% (83/2371) were infected from the blood of DP-treated children (p = 0.06).

#### ***Artemether-lumefantrine versus other artemisinin combination therapy***

The effects of AL on gametocyte carriage and malaria transmission to mosquitoes have also been compared with those of other ACT. Key findings are summarized in Table 3[31,33,36,40,63,65,69,70] (and Additional file 4[31,33,36,40,63,65,69,70]) and discussed below. For some ACT, the findings have to be interpreted with some caution since resistance to partner drugs (e g. AQ, sulphadoxine-pyrimethamine [SP]) will likely affect gametocyte clearance time after administration.

**Table 3:** **Effects of artemether-lumefantrine *versus *** **dihydroartemisinin-piperaquine and other artemisinin-based combination therapy on gametocyte carriage/clearance**

**Reference**	**Study description (location)**	**Study population**	**Gametocyte diagnostic method**	**Key conclusions**
4ABC Study Group [31]	Randomized head-to-head comparison (AL *vs* AQ + AS *vs* DP *vs* CD + A) in 7 sub-Saharan African countries	4,116 children (aged 6–59 months)	Microscopy	Gametocyte prevalence during follow-up was significantly lower and carriage time significantly shorter in children who received AL than in those treated with DP, AQ + AS, or CD + A
Faye *et al.*[36]	Multisite, randomized, open-label phase IV study in Dakar, Senegal, Ivory Coast	322 patients (aged >7 years)	Microscopy	Anti-gametocyte activity was more effective and rapid during treatment with AL than AS + AQ
	AL *vs* AS + AQ			
Tshefu *et al.*[33]	Phase III, parallel-group, double-blind, randomized, non-inferiority trial at 7 sites in Africa and 3 sites in Southeast Asia	1,272 adults and children	Microscopy PCR	Fixed-dose P-AS showed high clinical and parasitological response rates and rapid parasite clearance
	(AL *vs* P-AS)			
Zwang *et al.*[40]	Systematic review of comparative and non-comparative clinical trials in sub-Saharan Africa (16 countries, 33 sites)	11,700 patients (AL administered to 1,319 patients at 11 study sites)	Microscopy	Compared with AS + AQ, the risk of appearance of gametocytes was higher and the carriage duration was longer with the non-ACT than with AL and DP ACT regimens
	(AL *vs* AS + AQ *vs* AQ *vs* CQ + SP *vs* AQ + SP *vs* DP *vs* AS + SP)			
van den Broek *et al*. [63]	Comparator study in Kindamba, Republic of Congo	298 children	Microscopy	AL was clinically more effective than AS + SP and AS + AQ in these children
	(AL *vs* AS + AQ *vs* AS + SP)			
van den Broek *et al.*[67]	Open-label, randomized, 3-arm efficacy trial in Chittagong, Bangladesh	364 adults and children	Microscopy	ACTs block the development of new gametocytes. This effect has potential implications for the transmission of *P. falciparum* malaria
	(AL *vs* MQ + AS *vs* CQ + SP)			
				In contrast, CQ + SP therapy does not affect gametocyte development
Mutabingwa *et al*. [65]	Randomized comparator trial in Muheza, Tanzania	1,717 children (aged 4–59 months)	Microscopy	Gametocyte prevalence at day 14 in the ACT groups was significantly reduced compared with presentation
	(AL *vs* AQ *vs* AQ + AS *vs* AQ + SP)			
				ACT combinations led to lower gametocyte carriage, suggesting lower infectiousness with these treatments than with other combinations
Koram *et al.*[69]	Comparator study in Hohoe and Navrongo, Ghana	168 children (<5 years)	Microscopy	The prevalence of gametocytaemia was highest within the SP group, in-line with evidence to suggest that using SP alone increases prevalence of gametocytes, with possible increase in malaria transmission
	(AL *vs* AS + AQ *vs* CQ *vs* SP)			
				Gametocyte prevalence was lowest with AL and AS + AQ ACT regimens
Lefèvre *et al*. [70]	Randomized, open-label, parallel group 4-week trial in Thailand	219 adults and children with multidrug-resistant *P. falciparum* malaria	Microscopy	Gametocyte clearance was more rapid with AL than MQ + AS in these children
	(AL *vs* MQ + AS)			

Further data on these studies can be found in Additional file 4.

A: artesunate; ACT: artemisinin-based combination therapy; AL: artemether-lumefantrine; AQ: amodiaquine; AS: artesunate; CD: chlorproguanil-dapsone; CQ: chloroquine; DP: dihydroartemisinin-piperaquine; MQ: mefloquine; OR: odds ratio; P: pyronaridine; PCR: polymerase chain reaction; SP: sulphadoxine-pyrimethamine.

In the first comparative trial of the efficacy of three combination therapies in Bangladesh, AL and MQ + AS ACT effectively prevented the development of gametocytes whereas chloroquine + sulphadoxine-pyrimethamine (CQ + SP) did not [67]. At 42 days of follow-up, 46% of patients in the CQ + SP group had gametocytes at one or more visits, compared with 0.8% of patients treated with MQ + AS and 2.5% of patients who received AL. Similarly, a large (n = 1,717) randomized, four-arm trial of children (aged four to 59 months) in Tanzania reported substantially fewer gametocytes in two artemisinin-containing combination groups (AL and AQ + AS) than in the non-artemisinin-containing AQ + SP combination group at day 14 [65]. Gametocyte prevalence at day 14 was significantly reduced from that recorded at patient presentation in the artemisinin groups, but not with the non-artemisinin-containing regimens: 6% with AL, 12% with AQ + AS, 26% with AQ + SP, and 19% with AQ alone.

In a randomized, open-label phase IV study, a reduction in gametocyte carriage was demonstrated in AL and AS + AQ treatment arms, although clearance was more rapid following AL than AS + AQ; gametocytes had disappeared by day 14 in the AL group and by day 21 in the AS + AQ group [36]. An earlier study of 298 children from the Republic of Congo reported a similar gametocyte carriage rate with AL, AS + AQ and AS + SP; an increase in gametocytes was observed during the first two days of treatment, but reduced during the four weeks of follow-up, from 8 to 1% in the AL group, from 23 to 3% in the AS + AQ group, and from 26 to 5% in the AS + SP group [63]. These findings are consistent with those from a study of 168 children (<five years) in Ghana; treatment with AL was associated with a similar gametocyte clearance rate as AQ + AS; gametocytaemia peaked on day 1 (7.7% for AQ + AS and 11.8% for AL) and declined to 2% for both ACT on days 7 and 14 [69].

A systematic review covering the periods February 1999 to December 2006 reported data from 11,700 patients from 16 countries at 33 sites in sub-Saharan Africa [40]. Comparative studies included 5,987 patients who received AS + AQ and 5,713 treated with other anti-malarials (AL was administered at 11 sites to 1,319 patients). The overall median clearance time was day 14 in patients treated with AS + AQ, but varied widely by site (day 1 to 28). Compared with AS + AQ groups, the risk of gametocyte appearance post-admission was significantly higher with AQ, CQ + SP and AQ + SP, lower with AL and DP and not different with AS + SP. The overall carriage rate was 57% shorter with AL compared with AS + AQ.

Further data are provided by a randomized trial conducted in >4,100 children (aged six to 59 months) from 12 sites in sub-Saharan Africa [31]. Microscopically determined gametocyte prevalence during follow-up was significantly lower in children who received AL than in those treated with DP, AQ + AS or chlorproguanil-dapsone-artesunate (CD + A) in this large head-to-head comparison of four ACT (AL, AQ + AS, DP and CD + A). Gametocyte carriage time was also significantly shorter with AL than with AQ + AS and DP in this trial. However, in a randomized phase III trial of fixed-dose pyronaridine (P)-AS *vs* AL in 1,272 children and adults from Africa and Asia, the rate of gametocyte clearance did not significantly differ between groups (mean gametocyte clearance time: 14.7 hours with P-AS and 25.2 hours with AL) [33].

#### ***Artemether-lumefantrine versus non-ACT***

Non-ACT, and particularly SP and CQ, appear to have only partial activity against immature gametocytes [61], resulting in a relatively large proportion of individuals who remain gametocyte-positive after treatment. Significant increases in gametocyte carriage have been reported in some studies following non-ACT treatment [58,72]. Compared with non-ACT, AL has been associated with significantly reduced gametocyte carriage [51,66], and reduced infectivity to mosquitoes [66] (Additional file 1[8,9,11-15,17-19,21-72]).

### Effects of screening and treatment of asymptomatic carriers on population gametocyte carriage

Large proportions of *P. falciparum* infections are asymptomatic in malaria-endemic countries. Asymptomatic carriers can carry high levels of gametocytes, resulting in an infectious parasite reservoir that can infect newly hatched mosquitoes. While the role of asymptomatic carriers in disease transmission is currently unclear, it follows that the identification and treatment of asymptomatic carriers could reduce this pool of parasites and thus reduce transmission of malaria. This hypothesis is supported by modelling studies. A modelling and simulation analysis of community screening and treatment of asymptomatic carriers with AL predicted that treatment should clear asymptomatic infection and transmission of disease if carried out in the period prior to the transmission season [78]. The short- and long-term impact of mass treatment strategies in different endemic transmission settings was explored using a previously developed mathematical model. The data indicated that in small populations, temporary elimination may be feasible with several rounds of mass treatment together with vector control [5].

A relatively small pilot project conducted in a rural area of southern Zambia also demonstrated that active case detection (using the rapid diagnostic test (RDT)) is feasible and can identify reservoirs of asymptomatic infection and gametocyte carriers [34]. A large controlled, cluster-randomized trial was therefore conducted to determine the impact of identification (through community screening using the RDT) and AL treatment of asymptomatic carriers of *P. falciparum* on the number of cases of symptomatic malaria over a 12-month period compared with no RDT or AL treatment [24]. A total of 14,000 patients from 18 villages in Burkina Faso participated in the trial that consisted of four community screening campaigns, the first three before the rainy season in which the RDT was used to identify asymptomatic carriers and who were treated with AL from day 1, and the fourth after the rainy season at approximately 12 months. Overall, 96.1% of the population in the intervention arm who consented to participation were tested by RDT. A significantly lower mean gametocyte carriage was observed at day 1 of campaigns 2 and 3 following AL treatment *vs* controls (0.7 *vs* 5.4%; p < 0.0001, and 0.5 *vs* 5.8%; p < 0.0001), indicating a reduction in the parasite reservoir. However, this effect was not sustained as there was little difference between intervention and control arms in gametocyte prevalence at day 1 of campaign 4 (4.9 *vs* 5.1%; NS). Therefore, the effect on gametocyte carriage did not translate into interruption of malaria transmission or reduction in symptomatic cases.

There are likely to be several reasons why the findings from this study do not reflect the earlier simulation analysis [78], including the dilution effect via infected vectors from surrounding villages not involved in the study and the high transmission intensity. In this respect, in a limited study of this type, it would be problematic to prevent the introduction of disease by population migration and the highly mobile mosquito vector. Indeed, over 60 villages that were not being studied were in close proximity to the communities receiving intervention. Another confounding factor that might explain the ability to determine a benefit associated with AL *vs* control might be the fact that all participants in the trial were provided with an ITN, regardless of the intervention they received.

Taking this all into account, it is still possible that mass treatment with a drug that has effects on gametocyte carriage could translate into tangible clinical benefits. However, any future studies investigating this further will need to consider the learnings from this study conducted in Burkina Faso.

### Challenges

One major challenge with the findings reviewed here is the ability to make direct comparisons between studies that have been conducted using different methodologies, at different time periods, in different populations, and from geographic regions that differ with respect to transmission intensity and patterns of drug resistance.

Added to the above challenges are difficulties in the interpretation of gametocyte data from older studies that are dependent on microscopy methods alone (see below), studies that differ in terms of the timing of sampling for analysis of gametocyte data (i e. pre-specified sampling points, that do not allow for fluctuations in infectiousness, *vs* sampling throughout the study), and studies that did not set out to determine gametocyte viability and are associated with independent gametocyte data analyses. Diagnostic tests for gametocytaemia have evolved over recent years; early gametocyte data were based solely on microscopy that was considered the gold standard in malaria diagnosis for many years, and numerous studies have relied solely on this method to evaluate gametocyte data. However, the development of highly sensitive and specific PCR-based methods, such as the QT-NASBA technique, has led to far superior gametocyte detection rates [11,26,52,79]; in the large controlled, cluster-randomized trial of asymptomatic carriers reviewed above, an eight-fold greater prevalence of gametocytes was detected following quantitative reverse transcription-PCR compared with microscopy at day 1 of campaign 4 [24]. As such, it is difficult to draw definitive conclusions regarding the potential impact of ACT on malaria transmission potential based on microscopy data alone. It is notable that in most (42/62; 68%) of the studies identified by this literature search and reviewed here, gametocyte data are based solely on microscopy. However, it is also worth noting that despite the use of different diagnostic approaches to estimate baseline gametocyte counts and a range of other factors affecting baseline gametocytaemia, the gametocytocidal effect of AL is proportionately consistent across studies.

One further challenge, which is currently only partially answered, is the viability and infectivity of gametocytes following treatment. The majority of the studies analysed, with the exception of three studies in two settings (western Kenya and the Gambia) [26,46,59,61,66], did not have a study component (i e. feeding assays) that evaluated the viability and infectivity of the gametocytes that appear post treatment. Nevertheless, the limited available data suggest that ACT, and AL treatment in particular, are able to reduce the infectiousness of blood samples to mosquitoes when compared with non-ACT regimens [26,66].

The transmission abilities of sub-microscopic gametocyte densities represents a further challenge that requires investigation. Notably, the relative contribution to malaria transmission appears to be similar for carriers with sub-microscopic and microscopic gametocytaemia [59], underlining the need for sensitive detection methods that do not rely solely on microscopy.

In a randomized study of Kenyan children (n = 528), QT-NASBA revealed a significant proportion of children with sub-microscopic gametocytaemia (four-fold higher than expected based on microscopy alone). Treatment with AL did not significantly reduce the infectious proportion of the population when compared with SP monotherapy, but the percentage of mosquitoes that became infected was lower (3.6%) following treatment with AL than SP (6.9%) [61]. The authors concluded that sub-microscopic gametocytaemia is common after treatment, and the effect of ACT is moderate and does not prevent post-treatment malaria transmission. There is also emerging evidence that extended parasite clearance time and residual sub-microscopic parasitaemia after ACT treatment increases transmission to mosquitoes and results in a higher risk of recurrent parasitaemia [22]. In this study of Kenyan children, individuals with residual parasitaemia following AL treatment had a two-fold longer duration of gametocyte carriage (p = 0.0007), were more likely to infect mosquitoes (p = 0.015) and to have microscopically detectable parasitaemia during follow-up (p < 0.001).

However, there may be conflicting factors in Kenya. For example, this country has a relatively high prevalence of HIV [80], relatively high mortality in children [81], almost omnipresent schistosomiasis co-infection in children [82], and declining malarial transmission in some areas [83]. In contrast, the Gambia, is a very different setting with, for example, lower rates of HIV [84]. In a study conducted in this country, AL completely prevented transmission [66] at a time (2002) when malarial transmission was considerably higher than it is today [85,86]. Further data are required, therefore, to determine conclusively whether AL can reduce transmission of malaria – and in which populations and settings. Data are also required to compare the transmission-blocking effects of AL *vs* other ACT.

Utilizing ACT such as AL within malaria eradication strategies is also associated with a number of other challenges, such as balancing potential increased use of ACT (e g, treatment of asymptomatic carriers) with rational use and avoidance of resistance development. In this regard, artemisinin resistance has been predicted to spread more rapidly with increasing use of ACT, including when used in mass drug administration programmes. However, these predictions have mainly been based on mathematical modelling [87,88]. These models suggest that combinations of interventions are most effective for successful elimination, and that adding primaquine to ACT reduces the likelihood of artemisinin resistance developing. Therefore, while it has been argued that multiple rounds of mass drug administration (e g, with an ACT and gametocytocidal agent) could potentially result in at least temporary elimination of malaria if used alongside vector control [5], the careful selection of anti-malarials for this use would be crucial in order to minimize the risk of drug selection pressure and development of resistance. In this regard, it is crucial to ensure that any drugs that are chosen for such a programme are administered appropriately to avoid suboptimal drug exposure, which could lead to breakthrough resistance. This issue is highlighted by a recent systematic review, which found that ACT adherence levels varied from <30% for general ACT use in Kenya, to up to 100% adherence to AL in Malawi [89].

### Potential contribution of artemether-lumefantrine to future malaria elimination strategies

It is widely accepted that ACT will have a key role to play in future malaria elimination strategies. The specific place of different ACT, including AL, is the subject of much ongoing research and will undoubtedly be dependent on different demographic and geographical scenarios, and include consideration of factors such as endemicity levels, transmission intensity, resistance patterns to partner drugs, availability of specific drugs and rapid, cost-effective diagnostic testing kits.

Much of the gametocyte data reviewed here indicate that AL does possess gametocytocidal properties; rapid reduction in gametocyte carriage has been reported in studies of almost 6,000 children and adults, and possible interruption of malaria transmission has been observed when combined with indoor residual spraying in studies involving >8,000 children and adults. Superior gametocyte clearance rates and reduced infectiousness to mosquitoes has been demonstrated with AL when compared with DP in studies of >2,000 children and adults, and when compared with other ACT in studies involving >18,000 children and adults.

These findings suggest that AL is a logical ACT option for use in future malaria elimination programmes, possibly combined with a further gametocytocidal agent, such as primaquine (see below) [21], or in combination with preventative measures such as vector control (e g, ITNs and long-lasting insecticide-treated nets) and potentially future malaria transmission-blocking vaccines. Based on the recent data of Sawa *et al.*[26], AL appears to be a valuable ACT to reduce community-wide transmission of *P. falciparum* malaria, especially in areas of lower endemicity, while DP may be best suited to the prevention of re-infections in areas of higher endemicity. However, the choice of which ACT to use – AL or DP – is not clear cut. A recent systematic review suggested that DP is superior to AL in preventing further parasitaemia in Africa, although PCR-adjusted treatment failure was below 5% for both ACT [90]. Additionally, DP appeared to have a longer prophylactic effect on new infections. Nevertheless, findings presented here indicate that AL is more effective than DP in terms of gametocyte clearance, and this is most evident in the first few weeks post-treatment. This benefit may outweigh the relatively shorter prophylactic effect of AL.

The gametocytocidal medicine primaquine may have an important role in a malaria elimination campaign. ACT is recommended for individuals of all ages to be used with a single dose of primaquine (0.25 mg base/kg) to treat *P. falciparum* malaria [1,16]. Ideally, a glucose-6-phosphate dehydrogenase (G6PD) test is required prior to initiating treatment with primaquine, but this may not be feasible in the majority of malaria-endemic countries [1]. A recent study confirmed that primaquine is effective in reducing gametocyte carriage [21], although there are still questions remaining with respect to use in areas of high or moderate transmission, optimum timing of administration and use in asymptomatic *vs* symptomatic infections. In spite of these outstanding questions, AL has also been associated with a good safety profile in G6PD-deficient individuals [45], suggesting a possible role for AL combined with primaquine in this setting. Additionally, based on available data, the gametocytocidal effect of AL may synergize with that of primaquine when used in combination [33]. However, this area requires further research, given that the efficacy of primaquine is CYP2D6 dependent [91].

## Conclusions

It is anticipated that AL (along with other ACT) will continue to play a vital role in the treatment of uncomplicated falciparum malaria [90]. Like other ACT, AL maintains high efficacy against the asexual stage of falciparum parasites, which largely reduces parasitaemia in a population through treatment. Based on the data reviewed here, the effect on asexual parasites is complemented by a pronounced reduction in gametocyte carriage in both symptomatic patients and asymptomatic carriers. Indeed, it appears that AL may be the most potent ACT in terms of clearing gametocytes.

Considering all of the above, AL may play an important role in the drive towards malaria elimination. However, there is currently incomplete evidence as to whether residual transmission persists after ACT. Additionally, while there is some evidence that country-wide implementation of AL can contribute to reducing transmission compared with non-ACT [68], this was not confirmed in a cluster randomized trial in an area of very high transmission intensity in Burkina Faso [24]. What is clear is that if AL is used widely to reduce transmission, then strategies should be sought to protect against AL drug resistance. The use of AL with single dose primaquine could be helpful but requires further evaluation. More data is needed to help to determine the level gametocyte infectivity post treatment and the potential role of AL in malaria elimination programmes, and to compare AL with other ACT, including DP.

## Abbreviations

ACT: Artemisinin-based combination therapy; AL: Artemether-lumefantrine; AS-AQ: Artesunate-amodiaquine; AS-MQ: Artesunate-mefloquine; AQ: Amodiaquine; AS: Artesunate; CD + A: Chlorproguanil-dapsone-artesunate; CQ: Chloroquine; DP: Dihydroartemisinin-piperaquine; G6PD: Glucose-6-phosphate dehydrogenase; HR: Hazard ratio; ITN: Insecticide-treated net; MQ: Mefloquine; P: Pyronaridine; PCR: Polymerase chain reaction; QT-NASBA: Quantitative real-time Nucleic Acid Sequence-based Amplification; RDT: Rapid diagnostic test; SP: Sulphadoxine-pyrimethamine; TS: Trimethoprim-sulphamethoxazole; WHO: World Health Organization.

## Competing interests

A large percentage of the clinical trials that involved AL prior to registration of this product were sponsored by Novartis who hold the patent for this product.

## Authors’ contributions

MM is responsible for the content of this review manuscript. He critically reviewed, revised and approved the content at each stage of development, and read and approved the final version for submission. The views reflected in this manuscript are those of MM and not his employer, EDCTP.

## Supplementary Material

Additional file 1 **The effects of artemether-lumefantrine on gametocyte carriage/malaria transmission.** Most to least recent publication date. Click here for file

Additional file 2 Effects of artemether-lumefantrine on gametocyte carriage/clearance - summary of key data. Click here for file

Additional file 3 **Effects of artemether-lumefantrine *versus* dihydroartemisinin-piperaquine on gametocyte carriage/clearance.** Click here for file

Additional file 4 **Effects of artemether-lumefantrine *versus* dihydroartemisinin-piperaquine other artemisinin-based combination therapy on gametocyte carriage/clearance.** Click here for file
