# Supplementary material for: A review of the effects of artemether-lumefantrine on gametocyte carriage and disease transmission
Source: Malar J. 2014 Jul 28;13:291. doi: 10.1186/1475-2875-13-291 (PMC4126813; doi:10.1186/1475-2875-13-291)
Supplement: Additional file 1 — The effects of artemether-lumefantrine on gametocyte carriage/malaria transmission. Most to least recent publication date. [file 1475-2875-13-291-S1.doc]

**Additional File 1** The effects of AL on gametocyte carriage/malaria transmission (most to least recent publication date) [8,9,11-15,17-19,21-72].

| **Author (year)** | **Study description (location)** | **Study population** | **Intervention** | **Gametocyte diagnostic method** | **Key data regarding gametocyte carriage/malaria transmission** | **Key conclusions regarding AL** |
| --- | --- | --- | --- | --- | --- | --- |
| Eziefula AC et al (2014) [21] | Randomised, double-blind, placebo-controlled, dose-ranging primaquine trial (Jinja district, Eastern Uganda) | Children aged 1–10 years with uncomplicated falciparum  malaria and normal G6PD enzyme function (n=468) | AL + placebo  or with 0.1 mg/kg, 0.4 mg/kg, or 0.75 mg/kg (WHO reference dose) primaquine base | Microscopy,  QT-NASBA* | - Gametocyte prevalence at enrolment was 22.6% (104/461) by microscopy and 81.8% (365/446) by QT-NASBA, and did not differ between treatment groups (p=0.91 for microscopy and p=0.42 for QT-NASBA) - A longer mean gametocyte circulation time was reported in the 0.1 mg/kg primaquine group (p=0.0012) and the placebo group (p<0.0001) than in the reference 0.75 mg/kg group | - 0.4 mg/kg primaquine has similar gametocytocidal efficacy as the reference 0.75 mg/kg primaquine dose - Although primaquine shortened the duration of gametocyte carriage, even the highest dose did not completely eliminate gametocytes |
| Kakuru A et al (2013) [12] | Open-label randomised controlled trial (Tororo district, Uganda) | Children (≥4 months) with uncomplicated malaria (n=351)  100 HIV-unexposed  203 HIV-exposed  48 HIV-infected | AL (one or two 20 mg/120 mg tab twice-daily for 3 days) or DP plus TS prophylaxis | Microscopy | - Prevalence of gametocytaemia on day of diagnosis was similar for DP (6.2%) and AL (5.4%) after adjusting for TS use, age, and time since the last episode - Of 25,767 blood smears obtained during malaria follow-up, gametocytes were detected in 766 (3%) by microscopy - DP associated with 85% increase (RR=1.85, p<0.0001) risk of gametocytaemia during follow-up vs. AL, after controlling for TS prophylaxis, age, and development of recurrent parasitaemia | - Rate of gametocyte clearance was more than 2-fold greater with AL than DP (HR 2.20; p<0.001) |
| [Beshir KB](http://www.ncbi.nlm.nih.gov/pubmed?term=Beshir KB%5BAuthor%5D&cauthor=true&cauthor_uid=23945376) et al (2013) [22] | Open-label randomised, controlled trial  (Mbita,  West Kenya) | Children (≥6 months to 10 years) with microscopically confirmed *P. falciparum* mono infection and Hb >5g/dL (n=298) | AL (half a tab (20/120 mg) in a 6‐dose regimen or DP (40 mg/320 mg) in a 3-dose regimen | *Pfs25* QT-NASBA | - Residual parasitaemia was detected by qPCR in 31.8% (95% CI 24.6–39.8) of the children on day 3 after initiation of treatment - Residual parasitaemia was associated with a 2-fold longer duration of gametocyte carriage (p=0.0007), higher likelihood of infecting mosquitoes (RR=1.95, 95% CI 1.17–3.24, p=0.015) and higher parasite burden in mosquitoes (incidence rate ratio 2.92, 95% CI 1.61–5.31, p<0.001) | - Increased gametocyte production is a valuable marker of slow parasite clearance after ACT - A longer duration of gametocyte carriage results in higher infectivity to mosquitoes |
| [Joseph D](http://www.ncbi.nlm.nih.gov/pubmed?term=Joseph D%5BAuthor%5D&cauthor=true&cauthor_uid=23399782) et al (2013) [23] | Exploratory study  (Tabora municipality, Tanzania) | Children (6–59 months) with fever or history of fever (n=1,126) | AL dispersible 20 mg/120 mg tab in a 6-dose regimen | Microscopy | - 6 (30%) of the 20 patients >36 months had gametocytes on day 0 - In 5 patients the gametocytes persisted up to day 7 after treatment - 1 patient showed an increase in gametocytes after treatment from 4/500 WBC gametocytes on day 0 to 68/500 WBC on day 2 followed by a decrease on subsequent days. No gametocytes were seen microscopically after day 14 | - The therapeutic efficacy of AL dispersible against uncomplicated *P. falciparum* malaria was high in the study area |
| Tiono AB et al (2013) [24] | Single-centre, controlled, parallel, cluster randomised study  (Saponé, Burkina Faso) | Children <5 years (n=6,817 intervention arm; n=7,258  control arm) | AL/AL dispersible (20 mg/120 mg) twice a day for 3 days | Microscopy qRT-PCR* | - A lower prevalence of gametocyte carriage was found in the intervention arm than the control arm at day 1 of campaigns 2 and 3 (0.7% vs. 5.4%; p<0.0001 and 0.5% vs. 5.8%; p< 0.0001), but little difference was seen at day 1 of campaign 4 at the end of the study (4.9% vs. 5.1%; p=0.7208) - Prevalence of gametocytes   at day 1 of campaign 4 as assessed by qRT-PCR was around 8-fold greater in both arms compared with microscopy (49.7% vs. 6.0% intervention; 47.3% vs. 5.4% control) | - Systematic screening and treatment of asymptomatic carriers at the community level can reduce gametocyte carriage in a population |
| Pareek A et al (2013) [25] | Randomised, double-blind, double-dummy,  comparative, multicentric, phase III study  (India) | Patients (18–65 years) with uncomplicated malaria (n=158) | AS 100 mg/  lumefantrine 480 mg or artemether 80 mg/ lumefantrine 480 mg | Microscopy | - Median gametocyte clearance time was 12 hours in both treatment groups. All patients showed gametocyte clearance by day 7 and remained gametocyte-free until day 28 - There was no significant difference between treatment groups with regard to the presence of gametocytes at 24, 48 and 72 hours | - Complete gametocyte clearance was observed on day 7 in both treatment groups |
| Sawa P et al (2013) [26] | Randomised, open-label trial  (Mbita,West Kenya)  Use of QT-NASBA and mosquito feeding assays | Children (aged 6 months to 10 years) with uncomplicated falciparum malaria (n=298) | AL (20 mg/120  mg) in a 6-dose regimen or DP (40 mg/320 mg) | QT-NASBA feeding assays | - Enrolment gametocyte prevalence was 9.7% and 71.3% by microscopy and QT-NASBA respectively and did not differ between treatment arms - Mean duration of gametocyte carriage was significantly shorter with AL than DP (5.5 vs. 15.3 days, respectively; p=0.001) - Time to gametocyte clearance was significantly shorter with AL then DP (HR 2.35) - Infection of mosquitoes was lower with blood from AL-treated subjects (1.9%) than DP-treated subjects (3.5%; p=0.06) | - AL was associated with a significantly shorter duration of gametocyte carriage, and a significantly shorter time to gametocyte clearance than DP - Malaria transmission to mosquitoes was significantly lower after AL treatment than after DP |
| [Roberts CH](http://www.ncbi.nlm.nih.gov/pubmed?term=Roberts CH%5BAuthor%5D&cauthor=true&cauthor_uid=23347669) et al (2013) [13] | London | Patients with *P. falciparum* malaria (n=773) | Artemisinin  CQ | Microscopy | - 94/196 (12%) patients harboured gametocytes at presentation and 102 (13%) developed gametocytaemia during treatment | - Artemisinin treatment was associated with a lower rate of post-treatment gametocytaemia |
| [Abuaku B](http://www.ncbi.nlm.nih.gov/pubmed?term=Abuaku B%5BAuthor%5D&cauthor=true&cauthor_uid=23173737) et al (2012) [27] | One-arm prospective study  (Ghana) | Children (6–59 months) with symptoms of malaria (n=175) | AL (20 mg/120  mg) in a 6-dose regimen | Microscopy | - Gametocytaemia was prevalent on day 2 post-treatment, at 10.5% (4/38). By day 3 this had dropped to 5.3% (2/38), and by day 7 to 0, remaining at this level throughout follow-up | - ACT remains efficacious in clearing gametocytes in Ghana - Activity against gametocytes has the advantage of potentially slowing down the transmission of resistant alleles |
| [Sowunmi A](http://www.ncbi.nlm.nih.gov/pubmed?term=Sowunmi A%5BAuthor%5D&cauthor=true&cauthor_uid=22261842) et al (2011) [15] | Ibadan, Oyo state, Nigeria | Children <156 months with asymptomatic *P. falciparum* malaria  (n=835) | AS+AQ; AL; AS or AS+MQ | Microscopy | - Gametocyte density at enrolment was significantly higher in the group treated with AS+AQ than other treatment groups - Independent risk factors for gametocytaemia were gametocytaemia at enrolment (adjusted OR 46.39; 95% CI 22.3–96.46; p<0.0001) and treatment with AS (adjusted OR 6.74; 95% CI 1.79–25.27; p=0.005) or AS+MQ (adjusted OR 9.66; 95% CI 2.87–32.46; p<0.0.0001) relative to other ACTs - Haematocrit <25% and parasitaemia <50,000/μl blood were associated with an increased risk of gametocytaemia | - ACTs modified the risk factors associated with gametocyte carriage after treatment |
| Gbotosho GO et al  (2011) [28] | Antimalarial  efficacy studies (Ibadan, Southwestern Nigeria) | Children aged 0.5–15 years (n=2,585) | CQ (pre-policy  change, i.e. up to 2004) or AL (at policy and post-policy change, i.e. 2005 and after) | Microscopy | - Following the implementation of AL in 2005, gametocyte carriage declined significantly from 12.4% in 2001 to 3.6% in 2010 (p<0.0001) - AL significantly shortened the duration of male gametocyte carriage in individual patients | - AL reduced the rate of gametocyte carriage in children with acute falciparum infections at presentation and shortened the duration of male gametocyte carriage after treatment |
| Gbotosho GO et al  (2011) [14] | Antimalarial efficacy studies involving AS or ACTs   - (Ibadan, Southwestern Nigeria) | Children aged 0.5–15 years (n=2,585) | AL (20 mg/120 mg);  AS 4 mg/kg over 3 days;  AS+AQ  50 mg co-formulated;  AS+MQ 25 mg/kg  with 270 mg | Microscopy* | - Age of <3 years, haemat­ocrit <25%, parasitaemia <50,000/μL blood and enrolment before 2009 were associated with increased risk for gametocyte carriage at presentation - Gametocyte clearance times were similar in both anaemic and non-anaemic children (2.1 vs. 2.4, p=0.5) | - The emergence of gametocytes was significantly more frequent in anae­mic compared with non-anaemic children, thus sug­gesting that anaemia significantly impacts the release of gametocytes into the circulation |
| Gbotosho GO et al  (2011) [29] | A non-compartment pharmacokinetic model study  (Ibadan,  Southwestern Nigeria) | Children | CQ 30 mg/kg over 3 days;  AQ 30 mg/kg over 3 days;  PS as 25 mg/kg;  COT as 25 mg/kg twice daily for 5 days;  AS as 28 mg/kg over 7 days;  AL 20/120 mg | Microscopy | - The gametocyte sex ratio in children who carried gametocytes at enrolment was 0.18 + 0.02 (SEM) and in the subset of 52 children in whom GMI and GMLI were evaluated was 0.31 + 0.03 (SEM) - Values of GMI and GMLI were similar for each drug treatment group, and were significantly correlated (r=0.9, p=0.001) and close to unity | - AS, AL, AS+AQ and the non-ACT, AQ+SP, had ratios <1 suggesting potential for reducing the chance of   mosquito infectivity   - Index ratios >1 for 4-aminoquinolines and antifolates suggested potential for increasing the chance of mosquito infectivity |
| Makanga M et al  (2011) [30] | Pooled analysis of 7 studies conducted between 1996–2007:  4 studies in Thailand,  2 studies in Africa,  1 study in Europe and non-endemic regions of Colombia (non-immune adult travellers) | Adults (n=647), children (n=1,332) | AL dosing based on body weight:  5–<15 kg, 1 tab per dose; 15–<25 kg, 2 tab per dose; 25–<35 kg, 3 tab per dose; ≥35 kg, 4 tab per dose | Microscopy | - The proportion of adult patients with *P. falciparum* gametocytes at baseline was 9.7% (58/596), which decreased to 4.2% (23/554) after day 7 - Among children, 45 (5.1%) of 877 patients had gametocytes at baseline and 8 (0.9%) of 846 patients had gametocytes after day 7 - Gametocyte carriage decreased markedly from baseline after AL administration | - AL showed high cure rates and rapid resolution of parasitaemia, fever, and gametocytaemia in adults and children |
| 4ABC Study Group (2011) [31] | Randomised head-to-head comparison (7 Sub-Saharan African countries) | Children (n=4,116) aged 6–59 months | AL (n=1,226; 5–14 kg, 1 tab per dose; 15–24 25 kg, 2 tab per dose; 25– 34 kg, 3 tab per dose; ≥35 kg, 4 tab per dose)  DP, AQ-AS, CD+A | Microscopy | - Gametocyte prevalence during follow-up was significantly lower in children who received AL than in those treated with DP (OR 0.79), AQ+AS (OR 0.72), or CD+A (OR 0.50) - Gametocyte carriage time was significantly shorter with AL than with AQ+AS (OR 1.39) and DP (OR 1.26) | - Gametocyte prevalence during follow-up was significantly lower and carriage time significantly shorter in children who received AL than in those treated with DP, AQ+AS, or CD+A |
| Bousema T et al (2011) [11] | Review | Children and adults (many studies reviewed) | AL, other ACTs and non-ACTs (many studies reviewed) | Discusses limitations of microscopy | - Epidemiology and infectivity of *P. falciparum* and *P. vivax* gametocytes - Submicroscopic detection of gametocytes - Sampling issues - Effects of antimalarials on gametocytemia |  |
| [Bousema T](http://www.ncbi.nlm.nih.gov/pubmed?term=Bousema T%5BAuthor%5D&cauthor=true&cauthor_uid=20497536) et al (2010) [17] | Trial 1: Mbita, Kenya; Trial 2: Mnyuzi, Tanzania | Children with uncomplicated malaria, 0.5–10 years (n=160) and 3–15 years (n=108) in the two trials | Trial 1: SP: 25 mg/kg S + 1.25 mg/kg P as single dose or in combination with AS: 4 mg/kg once daily for 3 days or AQ AQ: 10 mg/kg once daily for 3 days  AL: 10 mg A + 60 mg L/5 kg twice daily for 3 days  Trial 2: Compared treatment with SP+AS with SP+AS followed by a single dose of primaquine (PQ: 0.75 m/kg once on the last [third] day of treatment) | Microscopy QT-NASBA | - Mean gametocyte circulation time was 6.5 days (95% CI 4.8–8.8) and 5.0 days (95% CI 4.2–6.1) in the non-ACT and ACT treated individuals, respectively (p=0.144) - Gametocyte density decreased sharply between days 0 and 3 (median reduction 99.1%, IQR 71.8–100%) - ACT reduced the duration of gametocyte carriage 4-fold to 13.4 days (95% CI 10.2–17.5) - Addition of PQ to ACT resulted in a further 4-fold reduction in duration of gametocyte carriage | - Artemisinins can shorten the duration of gametocyte carriage approximately 4-fold - PQ is a more potent gametocytocidal drug than artemisinins and led to a 4-fold reduction in the duration of gametocyte carriage compared with ACT alone |
| [Smithuis F](http://www.ncbi.nlm.nih.gov/pubmed?term=Smithuis F%5BAuthor%5D&cauthor=true&cauthor_uid=20832366) et al (2010) [32] | Open label randomized trial  (Rakhine state, Kachin state, and Shan state in Myanmar) | Patients aged 1–>14 years with uncomplicated malaria (n>800) | AS+MQ (total 12 mg/kg) plus MQ 25 mg base/kg on day 0 (n=161)  AS+MQ- hydrochloride 25 mg + 55 mg or 100 mg plus 220 mg tab  AL (20/120 mg) (n=169); DP (40/320 mg) (n=161)  AS+AQ 4 mg/kg per day plus 10.8 mg base/kg per day (n=162) | Microscopy | - Gametocyte carriage was variable following treatment with different ACTs, although all rates were higher with DP than other ACT regimens, including AL - The addition of primaquine reduced gametocyte carriage by around 12-fold for all ACT regimens | - All primaquine-containing treatment regimens had low gametocyte carriage (about 12-times lower than without primaquine) |
| [Tshefu AK](http://www.ncbi.nlm.nih.gov/pubmed?term=Tshefu AK%5BAuthor%5D&cauthor=true&cauthor_uid=20417857) et al (2010) [33] | Phase III, parallel-group, double-blind, randomised, non-inferiority trial  (7 sites  in Africa and 3 sites in Southeast Asia) | Children and adults (aged 3–60 years) with uncomplicated malaria (n=1,272) | 180 mg pyronaridine and 60 mg AS  AL 20/120mg | Microscopy  PCR | - Rate of gametocyte clearance did not significantly differ between groups (p=0.444). - The proportion of patients with gametocytes was highest on day 1 in both groups (P-AS 11.3%; AL 6.7%) - Mean gametocyte clearance time was 14.7 hours (SD 11.7) in the P-AS group and 25.2 hours (SD 17.2) in the AL group | - Fixed-dose P-AS showed high clinical and parasitological response rates and rapid parasite clearance |
| [Stresman GH](http://www.ncbi.nlm.nih.gov/pubmed?term=Stresman GH%5BAuthor%5D&cauthor=true&cauthor_uid=20920328) et al  (2010) [34] | Choma and Namwala districts, South Zambia | Patients aged 0–77 years (n=186 from 23 case homesteads and n=141 from 24 control homesteads) | AL (20/120 mg) | Microscopy RT-PCR* | - A gametocyte prevalence of 2.3% was found by active case detection (RDT) | - Using RDT results instead of PCR may be able to identify asymptomatic populations, particularly gametocytes |
| Okafor HU et al (2010) [35] | Prospective study (Enugu state, Southeast Nigeria; high level CQ resistance) | Children aged 6–120 months with uncomplicated malaria (n=139) | AQ (10 mg/kg) -  Sulfalene/pyrimethamine (ASP) (25 mg/kg);  AL  (1 tab/15 kg,  2 tab/16–24 kg, 3 tab/25–35 kg, 4 tab/>35 kg) | Microscopy | - 25/139 patients (17.98%) had gametocytaemia and of these 15 were detected on day 0; the remainder were found on later days - Gametocyte carriage at enrolment and by end of follow-up was similar in both treatment groups (p>0.05) - The difference between arms in gametocyte carriage by day 28 was not significant (p=0.49) | - Gametocyte carriage was similar in both treatment groups by end of study, although there was an increase in the ASP group initially |
| [Faye B](http://www.ncbi.nlm.nih.gov/pubmed?term=Faye B%5BAuthor%5D&cauthor=true&cauthor_uid=20214761) et al (2010) [36] | Multi-site, randomised, open-label phase IV study  (Dakar, Senegal, Ivory Coast) | Patients aged >7 years with uncomplicated malaria (n=322) | AQ (300 mg for children; 600 mg for adults)  AS (100 mg for children and 200 mg for adults) | Microscopy | - At presentation, 3% and 3.1% of the AL and AS+AQ groups, respectively, carried gametocytes - There was a gradual elimination of gametocytes in both arms, but this was more effective and rapid during AL treatment than AS+AQ - Gametocytes disappeared by day 14 in the AL group and by day 21 in the AS+AQ group | - Anti-gametocyte activity was more effective and rapid during treatment with AL than AS+AQ |
| [Assefa A](http://www.ncbi.nlm.nih.gov/pubmed?term=Assefa A%5BAuthor%5D&cauthor=true&cauthor_uid=20051120) et al (2010) [37] | 28 day therapeutic efficacy study  (Kersa district, Addis Ababa) | Patients with uncomplicated malaria (n=90) | AL 20/120 mg | Microscopy | - Gametocyte carriage was seen in 9% of patients <5 years, 7.3% in children, and 0 in those ≥15 years - Gametocyte clearance declined early in treatment (72.5% had cleared on day 1) - The remaining gametocyte load (28%) was maintained up to day 3 irrespective of treatment - Total clearance was observed on day 7 | - The study showed a rapid decline in gametocytes with treatment - The clearance rate was more rapid than that found in other studies, which reported the presence of gametocytes up to day 14 and beyond |
| Sowunmi A et al (2010) [38] | Ibadan, West Nigeria | Children with falciparum malaria (n=2,752) |  | Microscopy | - 10% (282/2,752) of the children had gametocytaemia on presentation - The majority of children without gametocytaemia at enrolment developed gametocytaemia on day 7 (36%, 156/433) - Gametocyte carriage within 2 weeks of commencing therapy was related to parasite clearance time; it increased from 13% (188/1,374) among children who cleared their parasitaemia on day 1 or 2 to 24.5% (245/1,000) among those who cleared their parasitaemia on day 3 or 4 (p<0.0001) | - A delay in parasite clearance was associated with   increased gametocyte carriage, and thus with potential for increased transmissibility of  drug-resistant phenotype |
| [Achan J](http://www.ncbi.nlm.nih.gov/pubmed?term=Achan J%5BAuthor%5D&cauthor=true&cauthor_uid=19622553) et al (2009) [39] | Randomised, open-label effectiveness study  (Uganda) | Children (n=175) | Quinine (10 mg/kg)  AL (20 mg /120 mg) | Microscopy | - Gametocytaemia was more common in the quinine group at day 7 compared with the AL group 14% (10/73) vs. 1% (1/74); p=0.001 - Total person time with gametocytes was 20 weeks for quinine compared with 5 weeks for AL (p<0.01) | - Gametocytaemia was more common in the quinine group compared with the AL group |
| [Zwang J](http://www.ncbi.nlm.nih.gov/pubmed?term=Zwang J%5BAuthor%5D&cauthor=true&cauthor_uid=19698172) et al (2009) [40] | Systematic review of comparative and non-comparative clinical trials conducted in Sub-Saharan Africa (16 countries, 33 sites) | 11,700 patients (AL administered to 1,319 patients at 11 study sites) | AS+AQ (based on body weight)  AS+AQ (based on age and weight range) | Microscopy | - There was no difference in clearance time between patients who had gametocytes on admission and those who developed gametocytaemia post-admission - The risk of gametocyte appearance post-admission compared to AS+AQ groups was lower with AL (p=0.01) and DP (p=0.001); higher with AQ (p=0.001), CQ+SP (p=0.001), and AQ+SP (p=0.001); and not different with AS+SP (p=0.288) - The overall carriage rate was 57% shorter with AL compared with AS+AQ | - Compared with AS+AQ, the risk of appearance of gametocytes was higher and the carriage duration was longer with the non-ACTs than with AL and DP ACT regimens |
| [Happi CT](http://www.ncbi.nlm.nih.gov/pubmed?term=Happi CT%5BAuthor%5D&cauthor=true&cauthor_uid=19075074) et al (2009) [41] | Ibadan, West Nigeria | Children aged <10 years with uncomplicated malaria (n=90) | AL  (1 tab/5­–14 kg; 2 tab/15–24 kg;  3 tab/25–34 kg; 4 tab/>34 kg) | PCR | - Gametocytes were detected in peripheral blood from 26 (29%) of 90 children treated with AL - The peak gametocyte density occurred 21 days post-treatment (144 gametocytes/µL of blood; n=5), although it was not significantly different (p=0.12) from the mean gametocyte density at enrolment (28 gametocytes/µL of blood; n=16) - There was an association (p=0.048) between treatment failure and the presence of gametocytes in patient samples before and after treatment - No *Pfmdr1* gene amplification was found in asexual-stage parasites or gametocytes in samples collected from patients either prior to or after treatment in patients with reoccurring infections | - The *Pfmdr1 N-F-D* haplotype may be a potential genetic marker of AL resistance, as demonstrated in this study, and there is a need to validate these markers in other areas   where the disease is endemic and where AL is currently being used   - *Pfmdr1* polymorphisms may result in reduction in the therapeutic efficacy of this newly adopted combination treatment for uncomplicated falciparum malaria in Saharan countries of Africa |
| [Sowunmi A](http://www.ncbi.nlm.nih.gov/pubmed?term=Sowunmi A%5BAuthor%5D&cauthor=true&cauthor_uid=19027703) et al (2009) [42] | Ibadan, Southwest Nigeria | Children (n=1,609) | CQ (10 mg/kg)  AQ (10 mg/kg)  SP (25 mg/kg)  COT (25 mg/kg)  AS (4 mg/kg)  SPP (20–25 mg/kg)  AL (20 mg)  LF (120 mg)  (1 tab/5–14 kg;  2 tab/15–24 kg;  3 tab/25–34 kg;  4 tab/>34 kg)  SP (25 mg/kg) | Microscopy | - Gametocyte carriage on presentation was 10% (162 children) - Non-ACT monotherapy significantly increased sex ratio producing a male-biased ratio, but ACT significantly reduced the sex ratio producing a female biased ratio - Pre-treatment sex ratio correlated negatively with haematocrit (r=−0.229, p=0.003) or gametocytaemia (r=−0.435, p<0.0001) but not with other clinical or parasitological parameters - Except for those treated with AS, higher frequencies of gametocyte densities were generally found in those treated with monotherapy | - Frequent occurrence of malaria-associated anaemia in children, its association with increased gametocyte carriage, and the proclivity of drug-related increased frequency of a sex ratio of 0.5 may have implications for transmission in endemic areas |
| [Bassat Q](http://www.ncbi.nlm.nih.gov/pubmed?term=Bassat Q%5BAuthor%5D&cauthor=true&cauthor_uid=19936217) et al (2009) [43] | Randomised, open-label, multicentre clinical trial (Burkina Faso, Kenya, Mozambique, Uganda and Zambia) | Children aged 6–59 months with uncomplicated malaria (n=1,553) | AL 20 mg/120 mg  (1 tab/5–14 kg;  2 tab/15–24 kg;  3 tab/25–34 kg)  DHA-PQP  (18 mg/kg) | Microscopy* | - Gametocyte prevalence at recruitment was similar in both study arms (ITT: DHA-PQP 11.75%; AL 12.94%, p=0.501; ePP: DHA-PQP 11.55%; AL 13.36%, p=0.326) - Gametocyte carriage measured as rate of person-gametocyte-weeks was significantly higher in the DHA-PQP group than in the AL group, both for the ITT (DHA-PQP: 43.97/1,000; AL: 21.43/1,000; p=0.005) and the ePP (DHA-PQP: 42.65/1,000; AL: 21.23/1,000; p=0.006) populations | - Patients treated with DHA-PQP had a significantly higher rate of person-gametocyte-weeks compared with those receiving AL |
| [Zwang J](http://www.ncbi.nlm.nih.gov/pubmed?term=Zwang J%5BAuthor%5D&cauthor=true&cauthor_uid=19649267) et al (2009) [44] | Individual patient data analysis of 7 open-label randomised comparative studies  (Northwestern Thailand,  Rakhine state, Myanmar, Southern Laos, and Western Cambodia) | Children and adults (n=3,547) | MQ+AS (MAS3) in Thailand, Myanmar, Laos and Cambodia  AL in Uganda  AQ+SP and AS+AQ in Rwanda | Microscopy | - Clearance of gametocytaemia was slower in DP groups than in the comparators, overall and in individual sites - At day 3, 7.4% of patients treated with DP (n=211) still had gametocytaemia vs. 1.8% of patients treated with AL (n=210) |  |
| Premji Z et al (2009) [45] | Randomised parallel-group, double-blind, double-dummy study  (South Africa) | Patients aged 1–15 years with acute uncomplicated malaria (n=1,372) | CDA (2/2.5/4 mg/kg/day)  AL 20 mg/120 mg  (1 tab/5–14 kg;  2 tab/15–24 kg;  3 tab/25–34 kg;  4 tab/>35 kg) | Microscopy | - Gametocytes were present at baseline in 25/912 (3%) patients in the CDA group and in 10/458 (2%) in the AL group - The proportion of gametocytaemic patients decreased similarly throughout the study in both treatment groups |  |
| [Oesterholt MJ](http://www.ncbi.nlm.nih.gov/pubmed?term=Oesterholt MJ%5BAuthor%5D&cauthor=true&cauthor_uid=19194499) et al (2009) [46] | Mbita,  West Kenya | Children aged 6 months to 10 years with uncomplicated malaria (n=528) | SP  SP+AQ  SP+AS  AL | Microscopy  *Pfs25* QT-NASBA  membrane-feeding assays | - Gametocyte prevalence at enrolment was 26.5% (50/189) by microscopy and 91.4% (118/129) by *Pfs25* QT-NASBA, and was not associated with number of *dhfr* mutations - The AUC (used for quantifying the transmission potential during follow-up of *Pfs25* QT-NASBA gametocyte density vs. time for non-ACT and ACT treated children) was significantly lower for ACT compared with non-ACT   treated children (p<0.001)   - The presence of all three mutations studied was not related to higher *Pfs25* QT-NASBA   gametocyte prevalence or density during follow-up, compared with double mutant infections, nor with the proportion of infected mosquitoes or oocyst burden | - Even at very high levels of SP resistance, the addition of AS to SP   monotherapy can have a beneficial effect on malaria transmission |
| [John CC](http://www.ncbi.nlm.nih.gov/pubmed?term=John CC%5BAuthor%5D&cauthor=true&cauthor_uid=19961670) et al (2009) [47] | Kipsamoite (7 villages) and Kapsisiywa (9 villages) in the  Nandi Hills district of Kenya | 8,094 adults and children | SP  co-artemether | - Microscopy - PCR* | - In 4 surveys of asymptomatic individuals during 2007–2008, a total of <0.3% were positive for *P. falciparum* trophozoites or gametocytes by microscopy during any period - In symptomatic individuals, gametocyte prevalence, assessed by microscopy, was low | - Treatment with AL (combined with IRS) reduced gametocyte carriage and density in children compared with the period prior to its implementation |
| [Sowunmi A](http://www.ncbi.nlm.nih.gov/pubmed?term=Sowunmi A%5BAuthor%5D&cauthor=true&cauthor_uid=19723589) et al (2009) [48] | Ibadan, Southwest Nigeria | Children with *P. falciparum* malaria (n=1,609) | Standard doses of CQ, AQ, SP,  SP+probenecid,  co-artesunate,  AL, AQ+AS,  AQ+SP | - Microscopy - PCR | - The frequency of a pre-treatment sex ratio of 0.5 was low (3%), and was significantly increased by non-ACT but not by artemisinin mono or combination drugs by day 7 (p=0.03 and p=0.44, respectively) |  |
| [Makanga M](http://www.ncbi.nlm.nih.gov/pubmed?term=Makanga M%5BAuthor%5D&cauthor=true&cauthor_uid=19818172), [Krudsood S](http://www.ncbi.nlm.nih.gov/pubmed?term=Krudsood S%5BAuthor%5D&cauthor=true&cauthor_uid=19818172) (2009) [49] | *Study A025:* randomised, double-blind study (Thailand)  *Studies A026 and A028:*  randomised, open-label studies  (Thailand)  *Study A2403:*  open-label non-comparative study | Children (>2 years) and adults (n=359) | AL |  | - *Study A025:* All patients cleared peripheral parasitaemia rapidly (median parasite clearance time = 44 hours). No patient developed gametocytaemia following treatment - *Study A2403:* None of the children had gametocytes after day 14 | - The efficacy of the 6-dose regimen of AL has been confirmed for consistently achieving 28-day PCR-corrected cure rates >95%, rapidly clearing parasitaemia and fever, and demonstrating a significant gametocytocidal effect, even in areas of widespread parasite resistance to other antimalarials |
| [Yeka A](http://www.ncbi.nlm.nih.gov/pubmed?term=Yeka A%5BAuthor%5D&cauthor=true&cauthor_uid=18545692) et al (2008) [50] | Randomised study (AL vs. DP) in Western Uganda (area of moderate transmission) | Children aged 6 months to 10 years with uncomplicated falciparum malaria (n=408 ) | AL – 20 mg/120 mg  (1 tab/5–14 kg;  2 tab/15–24 kg;  3 tab/25–34 kg;  4 tab/>35 kg)  DP (6.4 and 51.2 mg/kg) | Microscopy | - Presence of gametocytes at day 0 was 5.6% vs. 9.1% in DP and AL groups, respectively - Risk of developing gametocytes after therapy was significantly higher in patients with recurrent parasitaemia compared with those without recurrent parasitaemia in both the AL (34% vs. 1%, p<0.0001) and DP (24% vs. 2%, p<0.0001) treatment arms | - Patients treated with DP had a lower risk of developing gametocytaemia than those treated with AL after therapy |
| [White NJ](http://www.ncbi.nlm.nih.gov/pubmed?term=White NJ%5BAuthor%5D&cauthor=true&cauthor_uid=19091042) (2008) [9] | Review |  | ACT |  | - ACT treatment (either AL, AS-SP, or AS-CQ) was associated with a significant reduction in: probability of being gametocytaemic on the day of transmission experiments (OR 0.20; 95% CI 0.16–0.26), transmission to mosquitoes by slide-positive gametocyte carriers (OR mosquito infection 0.49; 95% CI 0.33–0.73), and AUC of gametocyte density (ratio of means 0.35; 95% CI 0.31– 0.41) - Partially effective antimalarial drug treatment increases gametocyte carriage both by reducing asexual parasite killing and by putting stress on the surviving asexual parasite population | - Mature *P. falciparum* gametocytes are drug-resistant and affected only by artemisinins and 8-aminoquinolines |
| [Sowunmi A](http://www.ncbi.nlm.nih.gov/pubmed?term=Sowunmi A%5BAuthor%5D&cauthor=true&cauthor_uid=18560227) (2008) [51] | Randomised clinical trial (Southwestern Nigeria) |  | AL 20 mg/120 mg  (1 tab/5–14 kg;  2 tab/15–24 kg;  3 tab/25–34 kg;  4 tab/>35 kg)  AQ  (1/2 tab/12 kg;  1 tab/12–20 kg;  1 tab/21–30 kg;  2 tab/31–50 kg;  3 tab/>50 kg) | Microscopy | - Clinical recovery from illness occurred in all children who carried gametocytes on treatment with AL and AQ-SP - Gametocytaemia was detected in 20 patients (11%) before treatment and in another 22 patients (12.2%) after treatment - Gametocyte carriage rates were similar in both combination treatment groups, but the AUC of gametocytaemia plotted against time was 8-fold higher in AQ-SP than in the AL-treated children - During follow-up, there was a short-lived but significant increase in the gametocyte sex ratio in children treated with AQ-SP but not in those treated with AL | - Artemisinin derivatives and artemisinin-based combinations reduce asexual parasitaemia quickly, but also reduce gametocyte carriage and density, and produce sex ratios that are less likely to favour transmissibility |
| [Mens PF](http://www.ncbi.nlm.nih.gov/pubmed?term=Mens PF%5BAuthor%5D&cauthor=true&cauthor_uid=19017387) et al (2008) [52] | Randomised study (AL vs. DP) in Mbita, Western Kenya  use of QT-NASBA | Children (n=146) | AL 20 mg/120 mg  DP 160 mg | Microscopy QT-NASBA | - At the start of the study, 3 patients in the DP arm (4.5%) and 6 patients in the AL arm (9%) had microscopically detectable gametocytes on day 7 - Persistence or development of gametocytes was significantly higher and longer at day 3, 7 and 14 in the DP group than AL arm, although after 28 days no difference could be observed between treatment arms QT-NASBA analysis on 56 DP-treated subjects and 54 AL-treated subjects detected considerably more gametocyte carriers at the start of the study compared with microscopy; 22 study subjects in the DP arm (39.3%) and 21 in the AL arm (38.9%) were harbouring gametocytes | - A more rapid reduction in gametocytes was observed with AL than with DP - QT-NASBA provides a far more sensitive method than microscopy in gametocyte detection |
| [Hommel M](http://www.ncbi.nlm.nih.gov/pubmed?term=Hommel M%5BAuthor%5D&cauthor=true&cauthor_uid=19090980) (2008) [8] | Mini review |  |  |  | - Artemisinins have broad-stage specificity in their antimalarial action, killing all asexual stages, as well as gametocytes, but have no effect on the exo-erythrocytic stages - Recent field studies have shown that artemisinin treatment significantly reduced gametocyte carriage compared with other drugs, an effect that was variable from one geographical area to another, depending on the level of malaria endemicity and access to treatment | - Artemisinin treatment significantly reduced gametocyte carriage compared with other antimalarial drugs |
| [Okell LC](http://www.ncbi.nlm.nih.gov/pubmed?term=Okell LC%5BAuthor%5D&cauthor=true&cauthor_uid=18613962) et al (2008) [19] | Raw data were pooled from 6 previously published randomised antimalarial trials  (Gambia and Kenya) | Patients (n=3,174) | CQ, SP  CQ+SP, SP+AQ;  CQ+AS, SP+AS1  SP+AS3,  AL | Microscopy QT-NASBA | - Compared with non-ACTs, ACTs significantly reduced the gametocyte AUC during follow up (ratio of means 0.35; 95% CI 0.31–0.41), gametocyte prevalence on day of feeding (OR 0.20; 95% CI 0.16–0.26), and transmission to mosquitoes by slide-positive gametocyte carriers (OR mosquito infection 0.49; 95% CI 0.33–0.73) - The reduction in mean gametocyte density in the ACT group compared with the non-ACT group varied over time during follow-up, being most marked up to day 7 and returning to non-significance by day 28 - AL had a significantly greater impact on gametocytaemia than the 3-dose regimens (CQ-AS and SP-AS) and was associated with lower transmission to mosquitoes - not statistically significant (p=0.460) - ACT impact on transmission to mosquitoes by slide-positive gametocyte carriers was similar among those with and without pre-treatment gametocytes - ACT impact on gametocytaemia was smaller if submicroscopic gametocyte carriers were taken into account; ratio of means of AUC comparing ACT to non-ACT = 0.40 (95% CI 0.23–0.69; p<0.001) using microscopy data, and 0.60 (95% CI 0.51–0.70; p<0.001) with QT-NASBA data | - All ACT treatment groups showed reduced gametocytaemia and a lower prevalence of infection among mosquitoes compared with any non-ACT group |
| [Juma EA](http://www.ncbi.nlm.nih.gov/pubmed?term=Juma EA%5BAuthor%5D&cauthor=true&cauthor_uid=19102746) et al (2008) [53] | Randomised, controlled, open-label study comparing AL tablets with AL paediatric suspension in West Kenya | Children aged 6–59 months with uncomplicated malaria (n=245) | AL  Group 1 (20/120 mg)  Group 2 (15/90 mg) | Microscopy | - 14 (11.2%) and 10 (8.2%) children in the AL tablets and AL suspension arms, respectively, had gametocytes on day 0 - Both treatments were effective in clearing gametocytes; only one patient in each arm had gametocytes on day 7, and 0 by day 28 | - AL tablets and the 3-dose suspension effectively cleared gametocytes in these children |
| [Hatz C](http://www.ncbi.nlm.nih.gov/pubmed?term=Hatz C%5BAuthor%5D&cauthor=true&cauthor_uid=18256423) et al (2008) [54] | Open-label, non-comparative study  (Europe and non-endemic regions of Colombia) | Non-immune adult travellers (n=165) | AL (20/120 mg) | Microscopy | - Between baseline and day 3, >20% of patients had *P. falciparum* gametocytes - No patient had gametocytes after day 7: 20.6% (days 0–3), 6.2% (days 4–7), 0 (days 8–42) | - Treatment with AL was effective in clearing gametocytes by end of study in non-immune adults |
| Sowunmi A et al (2007) [55] | Ibadan , Southwestern  Nigeria | Patients <10 years with uncomplicated malaria (n=181) | AL (20 mg/120 mg)  SP (500 mg/25 mg)  AQ (200 mg of base) | Microscopy* | - Despite significantly faster parasite clearance in the AL treated children, gametocyte carriage after treatment was similar to that in those treated with ASP |  |
| [Ibrahium AM](http://www.ncbi.nlm.nih.gov/pubmed?term=Ibrahium AM%5BAuthor%5D&cauthor=true&cauthor_uid=17244406) et al (2007) [56] | Eastern Sudan | Febrile patients (n=95) | AS (4 mg/kg)  SP (25 mg/kg)  AQ (10 mg/kg) | Microscopy* | - Gametocytes were detected in the blood smears of 3 patients, 1 subsequently given AS-SP and 2 subsequently given AS-AQ - No gametocytes were observed, in any patient during follow-up | - No gametocytes were observed at end of follow-up period after treatment with AS-SP and AS-AQ |
| [Kamya MR](http://www.ncbi.nlm.nih.gov/pubmed?term=Kamya MR%5BAuthor%5D&cauthor=true&cauthor_uid=17525792) et al (2007) [57] | Randomised single-blinded study (AL vs. DP) in Apac, Uganda (area of high transmission) | Children aged 6 months to 10 years with uncomplicated malaria (n=417) | AL 20/120  DP 40/320 | Microscopy | - Presence of gametocytes at day 0 was 19% vs. 26% in the DP and AL groups, respectively - Both treatments produced rapid clearance of parasitaemia with no parasites detected by day 3 - The risk of recurrent parasitaemia due to possible recrudescence (adjusted by genotyping) was significantly lower for participants treated with DP than with AL after 28 days | - Patients treated with DP had a lower risk of recurrent parasitaemia due to non-falciparum species, and development of gametocytaemia compared with patients treated with AL |
| [Thapa S](http://www.ncbi.nlm.nih.gov/pubmed?term=Thapa S%5BAuthor%5D&cauthor=true&cauthor_uid=17827354) et al (2007) [58] | Open-label, randomised, parallel-group efficacy  and safety study  (Nepal) | Patients (n=99) | SP (500/25 mg)  Coartem (20/120 mg) | Microscopy | - There was a significant rise in the percentage of patients who were slide positive for *P. falciparum* gametocytes after SP therapy to a peak on day 7, whereas the prevalence of gametocytaemia fell progressively after treatment with AL | - Gametocytaemia developed or emerged in a substantially greater proportion of patients allocated to SP than AL therapy |
| [Schneider P](http://www.ncbi.nlm.nih.gov/pubmed?term=Schneider P%5BAuthor%5D&cauthor=true&cauthor_uid=17360869) et al (2007) [59] | Mbita, Suba district, Western Kenya | *P. falciparum* malaria infected children (n=100) |  | Real-time *Pfs25* mRNA QT-NASBA  membrane feeding assays | - At the time of membrane feedings, 25% (25/100) of the children harboured microscopically-detectable gametocytes, and 75% (75/100) were gametocyte positive by *Pfs25* QT-NASBA - 62% (62/100) of children were infectious to mosquitoes, of whom 69% (43/62) had no microscopically-detectable gametocytes - Compared with children with microscopically detectable gametocytes, the proportion of infectious children was lower in children with submicroscopic gametocytaemia and lowest in children who were negative with both microscopy and *Pfs25* QT-NASBA (p=0.001) - 70% (35/50) of children with submicroscopic gametocytaemia were capable of infecting at least one mosquito | - The relative contribution to malaria transmission was similar for carriers with submicroscopic and microscopic gametocytemia |
| [Guthmann JP](http://www.ncbi.nlm.nih.gov/pubmed?term=Guthmann JP%5BAuthor%5D&cauthor=true&cauthor_uid=16837721) et al (2006) [60] | Caala, Central Angola | Children (n=138) | AS-AQ (4 mg/kg)  AL (20/120 mg) | Microscopy* | - After 28 days, there were 2/61 (3.2%) recurrent parasitaemias in the AL group and 4/64 (6.2%) in the AS-AQ group (p=0.72) - Only 1 (1.5%) patient (in the AS-AQ group) had gametocytes on day 28 vs. 5 (7.3%, AL) and 3 (4.3%, AS-AQ) at baseline |  |
| [Bousema JT](http://www.ncbi.nlm.nih.gov/pubmed?term=Bousema JT%5BAuthor%5D&cauthor=true&cauthor_uid=16544256) et al (2006) [61] | Kenya | Patients (n=528) | SP (25/1.25 mg)  SP + 4 mg/kg AS  SP + 10 mg/kg AQ  AL (20/120 mg) | - Microscopy - *Pfs25* QT-NASBA* - Membrane-feeding assays | - Gametocyte prevalence by *Pfs25* QT-NASBA was much higher than by microscopy - Gametocyte prevalence by *Pfs25* QT-NASBA was 89.4% (219/245) at enrolment and decreased after treatment with SP+AS, SP+AQ, and AL - During follow-up, gametocyte prevalence by microscopy was significantly lower among the children in the SP+AQ, SP+AS, and AL arms than among the children in the SP monotherapy arm - Membrane-feeding assays for a group of randomly selected children revealed that the proportion of infectious children was as much as 4-fold higher than expected when based on microscopy | - Children treated with AL had the lowest gametocyte prevalence by *Pfs25* QT-NASBA compared with the other treatments |
| [Chanda P](http://www.ncbi.nlm.nih.gov/pubmed?term=Chanda P%5BAuthor%5D&cauthor=true&cauthor_uid=16938133) et al (2006) [62] | Open-label one-arm prospective evaluation of paediatric suspension of AL in Zambia | Children <10 kg (n=91) | Coartesiane® artemether (180 mg/60 mL) and lumefantrine (1,080 mg/60 mL) oral suspension | Microscopy | - Gametocytes present on day 0 (368/µL blood) and day 2 (336/µL) reduced significantly by day 7 (80/μL), with none recorded on day 21 or day 28 | - AL paediatric suspension was associated with a significant and rapid reduction in gametocytes |
| [van den Broek I](http://www.ncbi.nlm.nih.gov/pubmed?term=van den Broek I%5BAuthor%5D&cauthor=true&cauthor_uid=17125496) et al (2006) [63] | Comparator study in Kindamba, Congo | Children with uncomplicated *P. falciparum* malaria (n=298) | AS+SP  AS+AQ | Microscopy | - The proportion of cases with gametocytes increased during the first 2 days of treatment, but decreased during the 4 weeks of follow-up, from 8–1% in the AL group, 23–3% in the AS+AQ group, and 26–5% in the AS+SP group | - AL was clinically more effective than AS+SP and AS+AQ in these children |
| [Makanga M](http://www.ncbi.nlm.nih.gov/pubmed?term=Makanga M%5BAuthor%5D&cauthor=true&cauthor_uid=16760509) et al (2006) [64] | Pooled analysis of 8 studies to compare 6-dose with 4-dose AL regimen  (4 studies in Africa,  4 studies in Thailand) | Children with uncomplicated *P. falciparum* malaria (n=544) | AL: 6 dose | Microscopy* | - The proportion of patients with gametocytes at early time-points (up to day 7) was lower for the 6-dose regimen than the 4-dose regimen, suggesting a rapid clearance of gametocytes, particularly with the 6-dose regimen, or clearance of merozoites before gametocyte formation occurred - Significantly fewer patients had circulating gametocytes at day 28 with the 6-dose than the 4-dose regimen | - The 6-dose regimen is associated with a more rapid clearance of parasites and a faster and more sustained reduction in gametocyte carriage than the 4-dose regimen |
| [Mutabingwa TK](http://www.ncbi.nlm.nih.gov/pubmed?term=Mutabingwa TK%5BAuthor%5D&cauthor=true&cauthor_uid=15850631) et al (2005) [65] | Randomised comparator trial (Muheza, Tanzania) | Children aged 4–59 months with uncomplicated malaria (n=1,717) | SP (1.25 mg/25 mg)  AQ (25 mg/kg)  AL (20/120 mg) | Microscopy* | - There were substantially fewer gametocytes at day 14 in the two ACT combination groups than in the AQ+SP group: AL 6%, AQ+AS 12%, AQ 19%, AQ+SP 26% | - Gametocyte prevalence at day 14 in the ACT groups was significantly reduced compared with presentation - ACT combinations led to lower gametocyte carriage, suggesting lower infectiousness with these treatments than with other combinations |
| [Sutherland CJ](http://www.ncbi.nlm.nih.gov/pubmed?term=Sutherland CJ%5BAuthor%5D&cauthor=true&cauthor_uid=15839740) et al (2005) [66] | Single-blind, open-label,  randomised, controlled trial  (Gambia) | Children with uncomplicated malaria (n=497) | CQ/SP (10 mg/12.5 mg)  AL (20/120 mg) | Microscopy* feeding assays | - Children treated with AL were significantly less likely to carry gametocytes within the 4 weeks following treatment than those receiving CQ/SP (30/378 [7.94%] vs. 42/86 [48.8%]; p < 0.0001) - Carriers in the AL group harboured gametocytes at significantly lower densities, for shorter periods (0.3 d vs. 4.2 d; p<0.0001) and were less infectious to mosquitoes at day 7 (p<0.001) than carriers who had received CQ/SP | - AL has specific activity against immature sequestered gametocytes, and has the capacity to minimise transmission of drug-resistant parasites |
| van den Broek IV et al (2005) [67] | Open-label, randomised, three arm  efficacy trial (AL vs. MQ+AS vs. CQ+SP)  Chittagong  Hill Tracts, Bangladesh | Patients aged >1 year with uncomplicated malaria (n=364) | CQ (10 mg/kg)  SP (25/1.25 mg)  MQ (15 mg/kg)  MQ + 4 mg/kg AS  AL (20/120 mg) | Microscopy | - Only 2% of patients had gametocytes on the day of admission, which increased at days 2 and 3 more markedly after CQ+SP compared with AL and MQ+AS - During the 42-day follow-up period, 46% of patients in the CQ+SP group vs. 2.5% and 0.8% and of patients treated with AL and MQ+ AS, respectively, had gametocytes at one or more visits | - ACTs block the development of new gametocytes. This effect has potential implications for the transmission of *P. falciparum* malaria - In contrast, CQ+SP therapy does not affect gametocyte development |
| [Barnes KI](http://www.ncbi.nlm.nih.gov/pubmed?term=Barnes KI%5BAuthor%5D&cauthor=true&cauthor_uid=16187798) et al (2005) [68] | Open-label in vivo study in KwaZulu-Natal province, South Africa, to determine therapeutic efficacy of a 6-dose regimen of AL | Patients aged >12 months with uncomplicated malaria (n=100) | AL (480/2,880 mg) | Microscopy | - During the 2,000 in vivo therapeutic efficacy study, 2/100 (2%) subjects for whom gametocyte densities were recorded were found to carry gametocytes after treatment with AL - AL was associated with a 95% decrease in gametocyte density among those carrying gametocytes | - AL contributed to a marked and sustained decrease in malaria cases, admissions, and deaths, by greatly improving clinical and parasitological cure rates and reducing gametocyte carriage |
| [Barnes KI](http://www.ncbi.nlm.nih.gov/pubmed?term=Barnes KI%5BAuthor%5D&cauthor=true&cauthor_uid=15878154), [White NJ](http://www.ncbi.nlm.nih.gov/pubmed?term=White NJ%5BAuthor%5D&cauthor=true&cauthor_uid=15878154) (2005a) [18] | Review  meta-analysis of RCT comparing ACTs and monotherapy |  |  |  | - In patients with no detectable gametocytaemia at baseline, the addition of 3 days AS dramatically reduced gametocyte carriage on day 7 (OR 0.11; 95% CI 0.09–0.15, n=2,734), with larger effects at days 14 and 28 (International Artemisinin Study Group, 2004) - ACTs decreased the transmission advantage of the resistant parasites over sensitive parasites, from a gametocyte carriage ratio of 4:1 (monotherapy resistant: sensitive) to a ratio of 1:1 (ACT resistant:sensitive) - The gametocyte-reducing effect of widespread use of a highly effective ACT (AS+MQ), has been shown to translate into a sustained 6-fold decrease in *P. falciparum* transmission in an area of low intensity transmission in Northwestern Thailand | - ACTs rapidly reduce both asexual and gametocyte stages of the *P. falciparum* lifecycle |
| [Koram KA](http://www.ncbi.nlm.nih.gov/pubmed?term=Koram KA%5BAuthor%5D&cauthor=true&cauthor_uid=16054584) et al (2005) [69] | Comparator study in Hohoe and Navrongo  Ghana | Children (<5 years) with uncomplicated malaria (n=168) | SP (25 mg/kg)  AQ (25 mg/kg) | Microscopy | - Gametocytaemia peaked on day 1 for AL (11.8%) and AS+AQ (7.7%) and declined to 2% for both regimens on days 7 and 14 - Prevalence of gametocytaemia remained highest within the SP group after day 1, and peaked on day 7 (38.5%) | - The prevalence of gametocytaemia   was highest within the SP group, in-line with evidence to suggest that using SP alone increases prevalence of gametocytes, with possible increase in malaria transmission   - Gametocyte prevalence was lowest with AL and AS+AQ ACT regimens |
| [Lefèvre G](http://www.ncbi.nlm.nih.gov/pubmed?term=Lefèvre G%5BAuthor%5D&cauthor=true&cauthor_uid=11463111) et al (2001) [70] | Randomised, open-label,  parallel group 4-week trial  (Thailand) | Adults and children with multidrug-resistant *P. falciparum* malaria (n=219) | - AL (20/120 mg) - MAS (54 mg/kg) - MQ (25 mg/kg) | Microscopy | - Nearly 10% of all patients had gametocytes detected on their pre-treatment slide - During the first 72 hours, gametocytes were detected in 26 (15.9%) patients taking AL and 10 (18.2%) taking MAS - The median time to gametocyte clearance was 72 hours for AL and 85 hours for MAS | - AL rapidly cleared gametocytes in multidrug-resistant *P. falciparum* malaria - Gametocyte clearance was more rapid with AL than MQ+AS in these children |
| [Looareesuwan S](http://www.ncbi.nlm.nih.gov/pubmed?term=Looareesuwan S%5BAuthor%5D&cauthor=true&cauthor_uid=10072142) et al (1999) [71] | Double-blind,  randomised trial  (Thailand) | Adult patients with *P. falciparum* malaria (n=252) | AL (20/120 mg)  MQ (250 mg) | Microscopy | - Overall, 62 (49%) of patients in the AL group and 74 (59%) of the patients receiving MQ had gametocytes in their blood at the beginning of the trial - The time to gametocyte clearance of the 136 evaluable patients was 152 hours for those receiving AL and 331 hours for those receiving MQ (p<0.001) - 45% of patients receiving AL achieved gametocyte clearance within 96 hours compared with only 18% of those receiving MQ | - AL showed a more rapid reduction in gametocyte numbers compared with MQ in this study population |
| [von Seidlein L](http://www.ncbi.nlm.nih.gov/pubmed?term=von Seidlein L%5BAuthor%5D&cauthor=true&cauthor_uid=9598454) et al (1998) [72] | Randomised, double-blind, controlled  efficacy trial (Gambia) | Children with uncomplicated falciparum malaria  (n=287) | AL (20/120 mg)  PS (12.5/250 mg) | Microscopy | - At 2 weeks of follow-up, 29% of the PS treated children but none of the AL-treated children carried gametocytes (p<0.0001) - Prior to therapy, gametocytes were detected in 4.3% of the children - Gametocytes were detected in 2.4% of children 3 days after treatment with AL and in none of the children 2 weeks after treatment - In contrast, the proportion of children with gametocytes increased following treatment with PS: 13.5% carried gametocytes 3 days after PS treatment and 29% 2 weeks after treatment | - AL results in fewer gametocyte carriers than PS in the children studied |

*Sampling for gametocyte data was undertaken in these studies only at specific time-points rather than throughout the course of the study.

A: artesunate; ACT: artemisinin-based combination therapy; AL: artemether-lumefantrine/co-artemether; AQ: amodiaquine; AS: artesunate; ASP: amodiaquine-sulfalene-pyrimethamine; AUC: area under the curve; CD: chlorproguanil-dapsone; CDA: chlorproguanil-dapsone-artesunate; CI: confidence interval; COT: cotrimoxazole; CQ: chloroquine; DHA: dihydroartemisinin; DP: dihydroartemisinin-piperaquine; ePP: enlarged per protocol; GMI: gametocyte male index; GMLI: gametocyte maleness load index; Hb: haemoglobin; HR: hazard ratio; IQR: interquartile range; IRS: indoor residual spraying; ITT: intention-to-treat; LF: lumefantrine; MAS: mefloquine + artesunate; MQ: mefloquine; mRNA: messenger ribonucleic acid; OR: odds ratio; P: pyronaridine; PCR: polymerase chain reaction; PQP: piperaquine; qPCR: quantitative polymerase chain reaction; qRT: quantitative reverse transcriptase; QT-NASBA: quantitative real-time Nucleic Acid Sequence-based Amplification; RDT: rapid diagnostic test; RR: relative risk; SEM: scanning electron micrograph; SP: sulphadoxine-pyrimethamine; SPP: SP + probenecid; RT: reverse transcriptase; tab: tablet; TS: trimethoprim-sulphamethoxazole; WBC: white blood cell; WHO: World Health Organization.
