# Supplementary material for: A review of the effects of artemether-lumefantrine on gametocyte carriage and disease transmission
Source: Malar J. 2014 Jul 28;13:291. doi: 10.1186/1475-2875-13-291 (PMC4126813; doi:10.1186/1475-2875-13-291)
Supplement: Additional file 2 — Effects of artemether-lumefantrine on gametocyte carriage/clearance - summary of key data. [file 1475-2875-13-291-S2.docx]

**Additional File 2**  **Effects of artemether-lumefantrine on gametocyte carriage/clearance - summary of key data [28,30,37,47,53,54,62,64,68,70]**

| **Reference** | **Study description** | **Study population** | **Gametocyte carriage/clearance data** | **Key conclusions** |
| --- | --- | --- | --- | --- |
| Makanga *et al.*  [30] | Pooled analysis of 7 studies conducted between 1996-2007:  4 studies in Thailand  2 studies in Africa  1 study in Europe and non-endemic regions of Colombia (non-immune adult travellers) | Pooled population:  647 adults and 1,332 children | - The proportion of adult patients with *P. falciparum* gametocytes at baseline was 9.7% (58/596), which decreased to 4.2% (23/554) after day 7 - Among children, 45 (5.1%) of 877 patients had gametocytes at baseline and 8 (0.9%) of 846 patients had gametocytes after day 7 - Gametocyte carriage decreased markedly from baseline after AL administration | - AL showed high cure rates and rapid resolution of parasitaemia, fever, and gametocytaemia in adults and children |
| [Gbotosho](http://www.ncbi.nlm.nih.gov/pubmed?term=Gbotosho%20GO%5BAuthor%5D&cauthor=true&cauthor_uid=22012222) *et al.* [28] | Anti-malarial efficacy studies in Ibadan, southwestern Nigeria | 2,585 children aged 0.5-15 years | - Following the implementation of AL in 2005, gametocyte carriage declined significantly from 12.4% in 2001 to 3.6% in 2010 (p<0.0001) - AL significantly shortened the duration of male gametocyte carriage in individual patients | - AL reduced the rate of gametocyte carriage in children with acute falciparum infections at presentation and shortened the duration of male gametocyte carriage after treatment |
| [Assefa](http://www.ncbi.nlm.nih.gov/pubmed?term=Assefa%20A%5BAuthor%5D&cauthor=true&cauthor_uid=20051120) *et al.* [37] | 28-day therapeutic efficacy study in Kersa District, Addis Ababa | 90 adults and children | - Gametocyte carriage was seen in 9% of patients <5 years, 7.3% in children, and 0 in those ≥15 years - Gametocyte clearance declined early in treatment (72.5% had cleared on day 1) - The remaining gametocyte load (28%) was maintained up to day 3 irrespective of treatment - Total clearance was observed on day 7 | - The study showed a rapid decline in gametocytes with treatment - The clearance rate was more rapid than that found in other studies, which reported the presence of gametocytes up to day 14 and beyond |
| [John](http://www.ncbi.nlm.nih.gov/pubmed?term=John%20CC%5BAuthor%5D&cauthor=true&cauthor_uid=19961670) *et al.* [47] | Kipsamoite (7 villages) and Kapsisiywa (9 villages) in the  Nandi Hills district of Kenya | 8,094 adults and children | - In 4 surveys of asymptomatic individuals during 2007–2008, a total of <0.3% were positive for *P. falciparum* trophozoites or gametocytes by microscopy during any period - In symptomatic individuals, gametocyte prevalence, assessed by microscopy, was low | - Treatment with AL (combined with IRS) reduced gametocyte carriage and density in children compared with the period prior to its implementation |
| [Hatz](http://www.ncbi.nlm.nih.gov/pubmed?term=Hatz%20C%5BAuthor%5D&cauthor=true&cauthor_uid=18256423) *et al.* [54] | Open-label, non-comparative study in Europe and non-endemic regions of Colombia | 165 non-immune adult travellers | - Between baseline and day 3, >20% of patients had *P. falciparum* gametocytes - No patient had gametocytes after day 7: 20.6% (days 0–3), 6.2% (days 4–7), 0 (days 8–42) | - Treatment with AL was effective in clearing gametocytes by end of study in non-immune adults |
| [Juma](http://www.ncbi.nlm.nih.gov/pubmed?term=Juma%20EA%5BAuthor%5D&cauthor=true&cauthor_uid=19102746) *et al.* [53] | Randomized, controlled, open-label study comparing AL tablets with AL paediatric suspension in Western Kenya | 245 children | - 14 (11.2%) and 10 (8.2%) children in the AL tablets and AL suspension arms, respectively, had gametocytes on day 0 - Both treatments were effective in clearing gametocytes; only 1 patient in each arm had gametocytes on day 7, and 0 by day 28 | - AL tablets and the 3-dose suspension effectively cleared gametocytes in these children |
| [Makanga](http://www.ncbi.nlm.nih.gov/pubmed?term=Makanga%20M%5BAuthor%5D&cauthor=true&cauthor_uid=16760509) *et al*. [64] | Pooled analysis of 8 studies to compare 6-dose with 4-dose AL regimen  4 studies in Africa  4 studies in Thailand | 544 children | - The proportion of patients with gametocytes at early time-points (up to day 7) was lower for the 6-dose regimen than the 4-dose regimen, suggesting a rapid clearance of gametocytes, particularly with the 6-dose regimen, or clearance of merozoites before gametocyte formation occurred - Significantly fewer patients had circulating gametocytes at day 28 with the 6-dose than the 4-dose regimen | - The 6-dose regimen is associated with a more rapid clearance of parasites and a faster and more sustained reduction in gametocyte carriage than the 4-dose regimen |
| [Chanda](http://www.ncbi.nlm.nih.gov/pubmed?term=Chanda%20P%5BAuthor%5D&cauthor=true&cauthor_uid=16938133) *et al.* [62] | Open label, one-arm prospective evaluation of paediatric suspension of AL in Zambia | 91 children (<10 kg) | - Gametocytes present on day 0 (368/µL blood) and day 2 (336/µL) reduced significantly by day 7 (80/μL), with none recorded on day 21 or day 28 | - AL paediatric suspension was associated with a significant and rapid reduction in gametocytes |
| [Barnes](http://www.ncbi.nlm.nih.gov/pubmed?term=Barnes%20KI%5BAuthor%5D&cauthor=true&cauthor_uid=16187798) *et al.* [68] | Open-label *in vivo* study in KwaZulu-Natal province, South Africa, to determine therapeutic efficacy of a 6-dose regimen of AL | 100 adults | - During the 2000 *in vivo* therapeutic efficacy study, 2/100 (2%) subjects for whom gametocyte densities were recorded were found to carry gametocytes after treatment with AL - AL was associated with a 95% decrease in gametocyte density among those carrying gametocytes | - AL contributed to a marked and sustained decrease in malaria cases, admissions, and deaths, by greatly improving clinical and parasitological cure rates and reducing gametocyte carriage |
| [Lefèvre](http://www.ncbi.nlm.nih.gov/pubmed?term=Lef%C3%A8vre%20G%5BAuthor%5D&cauthor=true&cauthor_uid=11463111) *et al.* [70] | Randomized, open-label,  parallel group 4-week trial in Thailand | 219 adults and children with multidrug-resistant *P. falciparum* malaria | - Nearly 10% of all patients had gametocytes detected on their pre-treatment slide - During the first 72 hours, gametocytes were detected in 26 (15.9%) patients taking AL - The median time to gametocyte clearance was 72 hours (3 days) | - AL rapidly cleared gametocytes in multidrug-resistant *P. falciparum* malaria |

AL: artemether-lumefantrine; IRS: indoor residual spraying.
