# Supplementary material for: A review of the effects of artemether-lumefantrine on gametocyte carriage and disease transmission
Source: Malar J. 2014 Jul 28;13:291. doi: 10.1186/1475-2875-13-291 (PMC4126813; doi:10.1186/1475-2875-13-291)
Supplement: Additional file 3 — Effects of artemether-lumefantrine versus dihydroartemisinin-piperaquine on gametocyte carriage/clearance. [file 1475-2875-13-291-S3.docx]

**Additional File 3**  **Effects of artemether-lumefantrine *versus* dihydroartemisinin-piperaquine** **on gametocyte carriage/clearance [12,26,32,44,50,52,57]**

| **Reference** | **Study description (location)** | **Study population** | **Gametocyte diagnostic method** | **Gametocyte carriage/clearance data** | **Key conclusions** |
| --- | --- | --- | --- | --- | --- |
| Kakuru *et al.* [12] | Open-label randomized controlled trial (AL *vs* DP+TS prophylaxis) in Tororo district, Uganda | 351 children (aged ≥4 months)  100 HIV-unexposed  203 HIV-exposed  48 HIV-infected | Microscopy | - Prevalence of gametocytaemia on day of diagnosis was similar for DP (6.2%) and AL (5.4%) after adjusting for TS use, age, and time since the last episode - Of 25,767 blood smears obtained during malaria follow-up, gametocytes were detected in 766 (3%) by microscopy - DP associated with 85% increased (RR=1.85, p<0.0001) risk of gametocytaemia *vs* AL during 28-day follow-up, after controlling for TS prophylaxis, age, and development of recurrent parasitaemia | - Rate of gametocyte clearance was more than 2-fold greater with AL than DP (HR 2.20; p<0.001) |
| Sawa *et al*. [26] | Randomized, open label trial (AL *vs* DP) in Mbita, western Kenya  Use of QT-NASBA and mosquito feeding assays | 298 children (aged 6 months to 10 years) | QT-NASBA  feeding assays | - Enrolment gametocyte prevalence was 9.7 and 71.3% by microscopy and QT-NASBA respectively and did not differ between treatment arms - Mean duration of gametocyte carriage was significantly shorter with AL than DP (5.5 *vs* 15.3 days, respectively; p=0.001) - Time to gametocyte clearance was significantly shorter with AL then DP (HR 2.35) - Infection of mosquitoes was lower with blood from AL-treated subjects (1.9%) than DP-treated subjects (3.5%; p=0.06) | - AL was associated with a significantly shorter duration of gametocyte carriage, and a significantly shorter time to gametocyte clearance than DP - Malaria transmission to mosquitoes was significantly lower after AL treatment than after DP |
| [Smithuis](http://www.ncbi.nlm.nih.gov/pubmed?term=Smithuis%20F%5BAuthor%5D&cauthor=true&cauthor_uid=20832366) *et al.* [32] | Open-label randomized trial (comparison of ACT, including DP *vs* AL) in Myanmar | >800 adults and children | Microscopy | - Gametocyte carriage was variable following treatment with different ACT, although all rates were higher with DP than other ACT regimens, including AL - The addition of primaquine reduced gametocyte carriage by around 12-fold for all ACT regimens |  |
| [Zwang](http://www.ncbi.nlm.nih.gov/pubmed?term=Zwang%20J%5BAuthor%5D&cauthor=true&cauthor_uid=19649267) *et al.* [44] | Analysis of 7 open-label randomized comparative studies (DP *vs* AL and *vs* other ACT) in northwest Thailand,  Rakhine state, Myanmar, southern Laos, and western Cambodia | 3,547 adults and children | Microscopy | - Clearance of gametocytaemia was slower in DP groups than in the comparators, overall and in individual sites - At day 3, 7.4% of patients treated with DP (n=211) still had gametocytaemia *vs* 1.8% of patients treated with AL (n=210) |  |
| [Yeka](http://www.ncbi.nlm.nih.gov/pubmed?term=Yeka%20A%5BAuthor%5D&cauthor=true&cauthor_uid=18545692) *et al.* [50] | Randomized study (AL *vs* DP) in Western Uganda (area of moderate transmission) | 408 children (aged 6 months to 10 years) | Microscopy | - Presence of gametocytes at day 0 was 5.6% *vs* 9.1% in DP and AL groups, respectively - Risk of developing gametocytes after therapy was significantly higher in patients with recurrent parasitaemia compared with those without recurrent parasitaemia in both the AL (34 *vs* 1%, p<0.0001) and DP (24 *vs* 2%, p<0.0001) treatment arms | - Patients treated with DP had a lower risk of developing gametocytaemia than those treated with AL after therapy |
| [Mens](http://www.ncbi.nlm.nih.gov/pubmed?term=Mens%20PF%5BAuthor%5D&cauthor=true&cauthor_uid=19017387) *et al.* [52] | Randomized study (AL *vs* DP) in Mbita, western Kenya, use of QT-NASBA | 146 children | Microscopy  QT-NASBA | - At the start of the study, 3 patients in the DP arm (4.5%) and 6 patients in the AL arm (9.0%) had microscopically detectable gametocytes on day 7 - Persistence or development of gametocytes was significantly higher and longer at day 3, 7 and 14 in the DP group than AL arm, although after 28 days no difference could be observed between treatment arms - QT-NASBA analysis on 56 DP-treated subjects and 54 AL-treated subjects detected considerably more gametocyte carriers at the start of the study compared with microscopy; 22 study subjects in the DP arm (39.3%) and 21 in the AL arm (38.9%) were harbouring gametocytes | - A more rapid reduction in gametocytes was observed with AL than with DP - QT-NASBA provides a far more sensitive method than microscopy in gametocyte detection |
| [Kamya](http://www.ncbi.nlm.nih.gov/pubmed?term=Kamya%20MR%5BAuthor%5D&cauthor=true&cauthor_uid=17525792) *et al.* [57] | Randomized single-blinded study (AL *vs* DP) in Apac, Uganda (area of high transmission) | 417 children (aged 6 months to 10 years) | Microscopy | - Presence of gametocytes at day 0 was 19 *vs* 26% in the DP and AL groups, respectively - Both treatments produced rapid clearance of parasitaemia with no parasites detected by day 3 - The risk of recurrent parasitaemia due to possible recrudescence (adjusted by genotyping) was significantly lower for participants treated with DP than with AL after 28 days | - Patients treated with DP had a lower risk of recurrent parasitaemia due to non-falciparum species, and development of gametocytaemia compared with patients treated with AL |

A: artesunate; ACT: artemisinin-based combination therapy; AL: artemether-lumefantrine; DP: dihydroartemisinin-piperaquine; HR: hazard ratio; OR: odds ratio; RR: relative risk: SD: standard deviation; TS: trimethoprim-sulphamethoxazole.
