# Supplementary material for: A review of the effects of artemether-lumefantrine on gametocyte carriage and disease transmission
Source: Malar J. 2014 Jul 28;13:291. doi: 10.1186/1475-2875-13-291 (PMC4126813; doi:10.1186/1475-2875-13-291)
Supplement: Additional file 4 — Effects of artemether-lumefantrine versus dihydroartemisinin-piperaquine other artemisinin-based combination therapy on gametocyte carriage/clearance. [file 1475-2875-13-291-S4.docx]

**Additional File 4**  **Effects of artemether-lumefantrine *versus* dihydroartemisinin-piperaquine other artemisinin-based combination therapy on gametocyte carriage/clearance [31,33,36,40,63,65,67,69,70]**

| **Reference** | **Study description (location)** | **Study population** | **Gametocyte diagnostic method** | **Gametocyte carriage/clearance data** | **Key conclusions** |
| --- | --- | --- | --- | --- | --- |
| 4ABC Study Group [31] | Randomized head-to-head comparison (AL *vs* AQ+AS *vs* DP *vs* CD+A) in 7 sub-Saharan African countries | 4,116 children (aged 6–59 months) | Microscopy | - Gametocyte prevalence during follow-up was significantly lower in children who received AL than in those treated with DP (OR 0.79), AQ+AS (OR 0.72), or CD+A (OR 0.50) - Gametocyte carriage time was significantly shorter with AL than with AQ+AS (OR 1.39) and DP (OR 1.26) | - Gametocyte prevalence during follow-up was significantly lower and carriage time significantly shorter in children who received AL than in those treated with DP, AQ+AS, or CD+A |
| [Faye](http://www.ncbi.nlm.nih.gov/pubmed?term=Faye%20B%5BAuthor%5D&cauthor=true&cauthor_uid=20214761) *et al.* [36] | Multisite, randomized, open-label phase IV study in Dakar, Senegal, Ivory Coast  AL *vs* AS+AQ | 322 patients (aged >7 years) | Microscopy | - At presentation, 3 and 3.1% of the AL and AS+AQ groups, respectively, carried gametocytes - There was a gradual elimination of gametocytes in both arms, but this was more effective and rapid during AL treatment than AS+AQ - Gametocytes disappeared by day 14 in the AL group and by day 21 in the AS+AQ group | - Anti-gametocyte activity was more effective and rapid during treatment with AL than AS+AQ |
| [Tshefu](http://www.ncbi.nlm.nih.gov/pubmed?term=Tshefu%20AK%5BAuthor%5D&cauthor=true&cauthor_uid=20417857) *et al.*  [33] | Phase III, parallel-group, double-blind, randomized, non-inferiority trial  conducted at 7 sites  in Africa and 3 sites in Southeast Asia  (AL *vs* P-AS) | 1,272 adults and children | Microscopy  PCR | - Rate of gametocyte clearance did not significantly differ between groups (p=0.444). - The proportion of patients with gametocytes was highest on day 1 in both groups (P-AS 11.3%; AL 6.7%) - Mean gametocyte clearance time was 14.7 hours (SD 11.7) in the P-AS group and 25.2 hours (SD 17.2) in the AL group | - Fixed-dose P-AS showed high clinical and parasitological response rates and rapid parasite clearance |
| [Zwang](http://www.ncbi.nlm.nih.gov/pubmed?term=Zwang%20J%5BAuthor%5D&cauthor=true&cauthor_uid=19698172) *et al.* [40] | Systematic review of comparative and non-comparative clinical trials conducted in Sub-Saharan Africa (16 countries, 33 sites)  (AL *vs* AS+AQ *vs* AQ *vs* CQ+SP *vs* AQ+SP *vs* DP *vs* AS+SP) | 11,700 patients (AL administered to 1,319 patients at 11 study sites) | Microscopy | - There was no difference in clearance time between patients who had gametocytes on admission and those who developed gametocytaemia post-admission - The risk of gametocyte appearance post-admission compared to AS+AQ groups was lower with AL (p=0.01) and DP (p=0.001); higher with AQ (p=0.001), CQ+SP (p=0.001), and AQ+SP (p=0.001); and not different with AS+SP (p=0.288) - The overall carriage rate was 57% shorter with AL compared with AS+AQ | - Compared with AS+AQ, the risk of appearance of gametocytes was higher and the carriage duration was longer with the non-ACT than with AL and DP ACT regimens |
| [van den Broek](http://www.ncbi.nlm.nih.gov/pubmed?term=van%20den%20Broek%20I%5BAuthor%5D&cauthor=true&cauthor_uid=17125496)  *et al*. [63] | Comparator study in Kindamba, Republic of Congo  (AL *vs* AS+AQ *vs* AS+SP) | 298 children | Microscopy | - The proportion of cases with gametocytes increased during the first 2 days of treatment, but decreased during the 4 weeks of follow-up, from 8 to 1% in the AL group, 23 to 3% in the AS+AQ group, and 26 to 5% in the AS+SP group | - AL was clinically more effective than AS+SP and AS+AQ in these children |
| van den Broek *et al.* [67] | Open-label, randomized, 3-arm  efficacy trial in Chittagong, Bangladesh  (AL *vs* MQ+AS *vs* CQ+SP) | 364 adults and children | Microscopy | - Only 2% of patients had gametocytes on the day of admission, which increased at days 2 and 3 more markedly after CQ+SP compared with AL and MQ+AS - During the 42-day follow-up period, 46% of patients in the CQ+SP group *vs* 2.5 and 0.8% of patients treated with AL and MQ+AS, respectively, had gametocytes at one or more visits | - ACTs block the development of new gametocytes. This effect has potential implications for the transmission of *P. falciparum* malaria - In contrast, CQ+SP therapy does not affect gametocyte development |
| [Mutabingwa](http://www.ncbi.nlm.nih.gov/pubmed?term=Mutabingwa%20TK%5BAuthor%5D&cauthor=true&cauthor_uid=15850631) *et al*. [65] | Randomized comparator trial in Muheza, Tanzania  (AL *vs* AQ *vs* AQ+AS *vs* AQ+SP) | 1,717 children (aged 4-59 months) | Microscopy | - There were substantially fewer gametocytes at day 14 in the two ACT combination groups than in the AQ+SP group: AL 6%, AQ+AS 12%, AQ 19%, AQ+SP 26% | - Gametocyte prevalence at day 14 in the ACT groups was significantly reduced compared with presentation - ACT combinations led to lower gametocyte carriage, suggesting lower infectiousness with these treatments than with other combinations |
| [Koram](http://www.ncbi.nlm.nih.gov/pubmed?term=Koram%20KA%5BAuthor%5D&cauthor=true&cauthor_uid=16054584) *et al.* [69] | Comparator study in Hohoe and Navrongo,  Ghana  (AL *vs* AS+AQ *vs* CQ *vs* SP) | 168 children (<5 years) | Microscopy | - Gametocytaemia peaked on day 1 for AL (11.8%) and AS+AQ (7.7%) and declined to 2% for both regimens on days 7 and 14 - Prevalence of gametocytaemia remained highest within the SP group after day 1, and peaked on day 7 (38.5%) | - The prevalence of gametocytaemia was highest within the SP group, in-line with evidence to suggest that using SP alone increases prevalence of gametocytes, with possible increase in malaria transmission - Gametocyte prevalence was lowest with AL and AS+AQ ACT regimens |
| [Lefèvre](http://www.ncbi.nlm.nih.gov/pubmed?term=Lef%C3%A8vre%20G%5BAuthor%5D&cauthor=true&cauthor_uid=11463111) *et al*. [70] | Randomized, open-label,  parallel group 4-week trial in Thailand  (AL *vs* MQ+AS) | 219 adults and children with multidrug-resistant *P. falciparum* malaria | Microscopy | - During the first 72 hours, gametocytes were detected in 26 (15.9%) patients in the AL arm and 10 (18.2%) in the MQ+AS arm - The median time to gametocyte clearance was 72 hours for AL and 85 hours for MQ+AS | - Gametocyte clearance was more rapid with AL than MQ+AS in these children |

A: artesunate; ACT: artemisinin-based combination therapy; AL: artemether-lumefantrine; AQ: amodiaquine; AS: artesunate; CD: chlorproguanil-dapsone; CQ: chloroquine; DP: dihydroartemisinin-piperaquine; HR: hazard ratio; MQ: mefloquine; OR: odds ratio; P: pyronaridine; PCR: polymerase chain reaction; QT-NASBA: quantitative real-time nucleic acid sequence-based amplification; SD: standard deviation; SP: sulphadoxine-pyrimethamine; TS: trimethoprim-sulphamethoxazole.
